# Preconditioning With Intermittent Hypobaric Hypoxia Attenuates Stroke Damage and Modulates Endocytosis in Residual Neurons

**DOI:** 10.3389/fneur.2021.750908

**Published:** 2021-12-15

**Authors:** Yaqi Wan, Lu Huang, Yanmin Liu, Weizhong Ji, Changxing Li, Ri-li Ge

**Affiliations:** ^1^Key Laboratory of Application and Foundation for High Altitude Medicine Research in Qinghai Province, Research Center for High Altitude Medicine, Qinghai University, Xining, China; ^2^Qinghai Provincial People's Hospital, Xining, China; ^3^Department of Neurology, The Second Affiliated Hospital of Guangzhou Medical University, Guangzhou, China; ^4^Department of Basic Medicine, Qinghai University, Xining, China

**Keywords:** hypobaric hypoxia, preconditioning, MCAO, neuroprotection, endocytosis

## Abstract

**Background:** Moderate hypobaric hypoxia induces cerebral ischemic tolerance. We investigated the optimal method for applying hypobaric hypoxia preconditioning at 5,000 m to ischemic brain tissue and combined it with proteomics to determine the mechanisms underlying this effect.

**Methods:** Male SD rats were randomly grouped as S (sham, *n* = 20), M (middle cerebral artery occlusion [MCAO], *n* = 28), H2M (intermittent hypobaric hypoxia preconditioned MCAO group, 2 h/day, 10 days, *n* = 20), H6M (intermittent hypobaric hypoxia preconditioned MCAO group, 6 h/day, 10 days, *n* = 28), and HpM (persistent hypobaric hypoxia preconditioned MCAO group, 10 days, *n* = 28). The permanent MCAO model was established based on the Zea Longa method. Infarction was assessed with the modified neurological severity score (mNSS) and 2,3,5-triphenyl tetrazolium chloride staining. The total protein expression of the neuron-specific nuclear protein (NeuN), cysteinyl aspartate specific proteinase 3 (caspase-3), cleaved-caspase-3, and interleukin 6 (IL-6) was determined using western blotting. We assessed the peri-infarct cortex's ultrastructural changes. A label-free proteomic study and western blot verification were performed on the most effective preconditioned group.

**Results:** The H6M group showed a lower infarct volume (*p* = 0.0005), lower mNSS score (*p* = 0.0009) than the M group. The H2M showed a lower level of IL-6 (*p* = 0.0213) than the M group. The caspase-3 level decreased in the H2M (*p* = 0.0002), H6M (*p* = 0.0025), and HpM groups (*p* = 0.0054) compared with that in the M group. Cleaved-caspase-3 expression decreased in the H2M (*p* = 0.0011), H6M (*p* < 0.0001), and HpM groups (*p* < 0.0001) compared with that in the M group. The neurons' ultrastructure and the blood-brain barrier in the peri-infarct tissue improved in the H2M and H6M groups. Immunofluorescence revealed increased NeuN-positive cells in the peri-infarct tissue in the H6M group (*p* = 0.0003, H6M vs. M). Protein expression of Chmp1a, Arpc5, and Hspa2 factors related to endocytosis were upregulated in the H6M compared with those of the M group (*p* < 0.05 for all) on western blot verification of label-free proteomics.

**Conclusions:** Intermittent hypobaric hypoxia preconditioning exerts a neuroprotective effect in a rat stroke model. Persistent hypobaric hypoxia stimulation exhibited no significant neuroprotective effect. Intermittent hypoxic preconditioning for 6 h/day for 10 days upregulates key proteins in clathrin-dependent endocytosis of neurons in the cortex.

## Introduction

Stroke is among the top three causes of death and one of the major causes of permanent disability worldwide (1), leading to tremendous social and economic burdens (2). Further, individuals who have suffered a first stroke are at high risk of recurrent strokes, thereby creating a life-long concern for patients (3). Tissue-plasminogen activator remains the only effective drug approved by the United States Food and Drug Administration for the treatment of strokes (4, 5). Since tissue-plasminogen activator is recommended to be administered within 4.5 h due to the relatively short half-life (3–4.5 min) (6, 7), many patients cannot receive timely and efficient treatment. Therefore, the prevention and treatment of stroke require further investigation. Numerous animal models have been used to examine potential preventative and therapeutic methods to reduce the severity of acute brain injuries (8, 9).

In the 1980's, it was suggested that sub-lethal stimulation induces ischemic tolerance to protect vital organs, such as the heart and brain, from lethal ischemic injury (10). Moreover, it was observed that patients with a history of transient ischemic attacks exhibited better clinical outcomes than those without an attack history (11–14). Severe hypoxia has long been regarded as detrimental to the human body and may be a key pathogenic factor in pulmonary hypertension (15, 16) and right ventricular hypertrophy (17, 18), even leading to dysfunction of the reproductive system in female rats (19). Studies have revealed correlations between high-altitude hypoxia and neurological disorders or diseases (cerebral edema, sleep disorder, cognition impairment, and cerebral vascular leakage) (20–23). Most harmful conditions reported in the literature are based on chronic (>2 weeks) or high-intensity (>5,500 m) hypoxic exposure (24–26). Moderate hypoxia (3,500–5,500 m) (27) is a feasible, economical, and effective non-invasive method of preconditioning that may enhance tissue tolerance through moderate stimulation aimed at achieving tissue protection. Numerous basic research efforts have demonstrated the neuroprotective effect of hypoxic preconditioning (HPC) from various perspectives (28–31). Intermittent hypobaric hypoxia was reported to improve vasodilation (32), reduce vascular sclerosis, increase left ventricular ejection fraction, and upregulate the expression of antioxidant enzymes in cardiac tissues (33). It may also be beneficial to the cardiovascular system. Additionally, the upregulation of heat shock protein 70 may play a pivotal role in tissue tolerance during the hypobaric hypoxia procedure (34, 35). Activation of hypoxia-inducible factor and its downstream target genes, including glucose transporters, vascular endothelial growth factor b, and erythropoietin, has also been demonstrated to contribute to HPC-induced ischemic tolerance (28, 36). Hypoxic preconditioned neural stem cells improve the efficacy of stem cell therapy in brain injury (37, 38). Given the controversy regarding preprocessing, we hypothesized that the protective effect of hypobaric hypoxia preconditioning (HHP) may coexist with the damage incurred; however, when the stimulation is too strong, more serious tissue damage occurs easily. To date, two main techniques (39) that could affect the neuroprotective function exist. These are as follows: persistent hypoxic preconditioning (PHP) and intermittent hypoxic preconditioning (IHP). Nevertheless, the optimal duration of intermittent hypoxia stimulation and the difference between the two preconditioning protocols remain unclear. Therefore, we aimed to examine these factors by establishing different preconditioned groups in a rat stroke model. Our findings contribute markedly to the study of ischemia tolerance and provide new insights regarding endogenous protection with implications for clinical drug therapy in cerebral ischemic diseases, such as stroke. Clathrin-dependent endocytosis (CDE) is the optimum internalization procedure of cell membrane receptors; almost all signal receptors function through CDE (40). It also represents the main pathway for transmembrane transporters and receptor uptake in both humans and animals. CDE functions in response to changes in the environment (41); fragment C of tetanus toxin is proven to enter the neurons through CDE and play a neuroprotective role in degenerative disease (42). Promotion of CDE could inhibit the damage caused due to glutamate neurotoxicity (43). Changes in atmospheric pressure and oxygen can also affect this biological process, and there may be an association between HHP and CDE.

## Materials and Methods

### Animals

A total of 104 Sprague Dawley rats (male, 7 weeks old, 180–220 g) were acquired from the Animal Center of Xi'an Jiao Tong University (Xi'an, China). All procedures were approved by the Ethics Committee for Animal Experimentation of Qinghai University according to the National Institutes of Health Guide for the Care and Use of Laboratory Animals (https://grants.nih.gov/grants/olaw/Guide-for-the-Care-and-use-of-laboratory-animals.pdf). All rodents used in the study were housed in a well-conditioned environment (18–22°C, 12-h light/dark cycle) and were provided food and water *ad libitum*.

### Experimental Design

**Figure 1:**
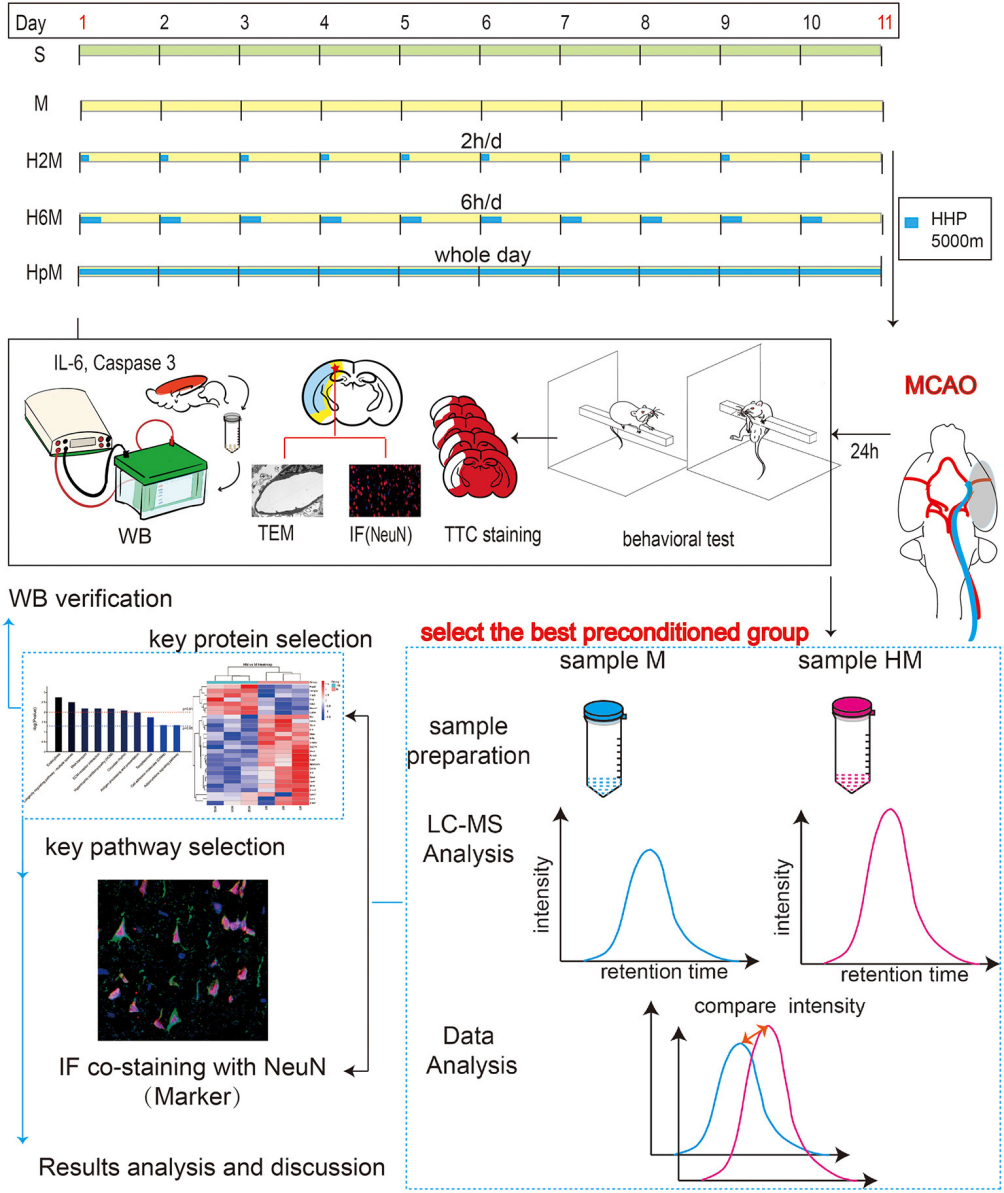
Experimental workflow. One hundred four rats were randomly divided into five groups: group S (sham, *n* = 20), group M (middle cerebral artery occlusion [MCAO], *n* = 28), group H2M (intermittent hypobaric hypoxia preconditioned MCAO group, 2 h/day, *n* = 20), group H6M (intermittent hypobaric hypoxia preconditioned MCAO group, 6 h/day, *n* = 28), and group HpM (persistent hypobaric hypoxia preconditioned MCAO group, *n* = 28). Behavioral tests and morphological staining (TTC staining) were used to analyze the severity of infarction. Total protein expression of NeuN (a specific marker of mature neurons), caspase-3, cleaved-caspase-3, and IL-6 was estimated using western blotting, which explained the severity of injury from different perspectives. Ultrastructural changes were observed under a transmission electron microscope. The most effective pretreatment group was selected for further label-free proteomic study and provided a reliable direction for mechanism exploration. Western blotting was used to verify the expression of the target protein, and key markers for the biological process were detected using immunofluorescence. caspase-3, cysteinyl aspartate specific proteinase 3; IL-6, interleukin 6; NeuN, neuron-specific nuclear protein; TTC, 2,3,5-triphenyl tetrazolium chloride.

The experimental workflow is presented in Figure 1 and illustrates the effect of HHP in ischemic rats. The rats (*n* = 104) were randomly divided into five groups: S (sham, *n* = 20), M (middle cerebral artery occlusion [MCAO], *n* = 28), H2M (intermittent hypobaric hypoxia preconditioned MCAO group, 2 h/day, 10 days, *n* = 20), H6M (intermittent hypobaric hypoxia preconditioned MCAO group, 6 h/day, 10 days, *n* = 28), and HpM (persistent hypobaric hypoxia preconditioned MCAO group, 10 days, *n* = 28). The permanent MCAO model was established based on the Zea Longa method in the four preconditioned groups. Twenty-four hours after the MCAO procedure, behavioral tests and morphological staining (using 2,3,5-triphenyl tetrazolium chloride) were performed to analyze the severity of the infarction; brain tissue was isolated for proteomics and western blotting. Total protein expressions of the neuron-specific nuclear protein (NeuN; a specific marker of mature neurons), cysteinal aspartate specific proteinase 3 (caspase-3), cleaved-caspase-3, and interleukin-6 (IL-6) were estimated using western blotting, which reflected the severity of injury from various perspectives. Ultrastructural alterations were evaluated using transmission electron microscopy. The most effective preconditioned group was selected for further label-free proteomic studies and provided a reliable direction for the exploration of a mediating mechanism.

### Surgery for the MCAO Model

The MCAO model was established as previously described (44). After weighing and induction of anesthesia (1% pentobarbital sodium, 35 mg/kg), animals were fixed onto an operating table in a supine position, and their neck region was shaved and disinfected. A midclavicular longitudinal incision (3 cm) was made. Arteries [left common carotid artery (CCA), left external carotid artery, internal carotid artery (ICA)] and nerves were fully exposed by separating the subcutaneous muscle tissue. The proximal ends of the external carotid artery and CCA were ligated. The ICA was clipped temporarily. A V-shaped incision was made in the CCA, and the artery clamp was released, allowing for the filament insertion (Doccol filament, 403556PK5Re, Sharon, USA) from the CCA into the ICA. Once resistance was encountered, the ICA was fixed, ligated, and plugged with a silk thread. According to the Zea Longa scoring system, a score of 1–3 points was considered as indicating a successful model whereas subarachnoid hemorrhage (anatomical study) or absence of neurological deficit symptoms (0 points) was considered as indicating a failed model. The S group received similar treatment as that to the M group except that no filament was inserted. All rats underwent rectal temperature monitoring to ensure that their bodies were maintained at 36.5 ± 0.5°C, intraoperatively.

### Model for HHP

In the preconditioned groups, rats were placed in a hypobaric hypoxia chamber (Dyc-3000; Guizhou Feng Lei Aviation Machinery Co., Ltd., Guangzhou, China) for different durations each day for 10 days and were exposed to hypoxia at a progressively increased simulated altitude of 5,000 m (atmospheric pressure: 52.93 kPa, temperature: 18°C, relative sea-level oxygen content: 10.24%). The H2M group received HHP stimulation for 2 h/day. The H6M group was maintained at the target altitude for 6 h/day. Rats in the HpM group were kept in a persistent hypoxic environment for 10 days.

### Evaluation of Infarct Size and Neurobehavioral Outcomes

Twenty-four hours after the occlusion, the neurological deficits of the permanent MCAO rats in four groups (M, H2M, H6M, and HpM) were evaluated randomly and in a double-blinded manner according to the modified neurological severity score (mNSS) (45) and the Longa score (46), which are widely used for behavioral assessment in rats after permanent cerebral ischemia. Neurological deficits, including limb motor function, balance, sensory (vision, tactus, and deep sensation), reflex absence, and abnormal movements, were estimated on a large scale. Each aspect of neural function was graded from 0 to 18 (0: normal score; 18: maximum damage). Therefore, the higher the score, the more serious the brain injury (47).

Five rats from each group were randomly selected for morphological staining. Freshly prepared brain slices (thickness: 2 mm) were incubated at 37°C in culture dishes containing the 2,3,5-triphenyl tetrazolium chloride (2%) stain. The formula to calculate the corrected infarcted volume percentage was as previously described (48).

Image-Pro plus 6.0. software was used to calculate the corrected percentage of cerebral infarction volume in each group.

### Transmission Electron Microscopy Analysis

Two rats from each group were randomly selected, perfused with normal saline, and fixed with glutaraldehyde. Cerebral cortex tissue (1 cm^3^) in the peri-infarct area was collected, and the samples were pre-fixed with glutaraldehyde (3%) and post-fixed with osmium tetroxide (1%). Acetone was used to gradually dehydrate the samples using a concentration gradient of 30, 50, 70, 80, 90% 95, and 100% (three changes in 100%). The dehydrated samples were successively treated with a mixture of a dehydrating agent, epoxy resin (Epon812), and osmotic solution at ratios of 3:1, 1:1, and 1:3; each step was performed for 30–60 min. The infiltrated sample block was first placed in an appropriate mold, and the embedding solution was poured into it. After polymerization, slices (~50 nm) were prepared using a microtome and transferred onto copper mesh grids. The samples were stained with uranium acetate and lead citrate for 15–20 min at room temperature (18–22°C) and observed using a JEM-1400 plus transmission electron microscope (JEOL Ltd., Tokyo, Japan).

### Evaluation of NeuN, IL-6, Caspase-3, and Cleaved-Caspase-3 Protein Levels in the Cortex

Expression levels of IL-6, caspase-3, and cleaved-caspase-3 proteins, extracted from the left hemisphere of the cerebral cortex of rats in the five groups, were assessed using western blotting. Equal quantities (10 μg) of the desaturated sample mixture from the five groups were added into different wells on 10% sodium dodecyl sulfate-polyacrylamide gel electrophoresis gels. Subsequently, the total protein was fully transferred onto polyvinylidene fluoride membranes (Millipore, Billerica, Massachusetts, USA). The membrane with total protein was blocked using bovine serum albumin (5%) for 2 h at 18–22°C and then incubated overnight with antibodies against NeuN (1:2000, ab177487, Abcam, Cambridge, MA, USA), beta-actin (1:2000, ab197277, Abcam), IL-6 (1:500, BA4339, Boster, Wuhan, China), or caspase-3 (1:500, BA3257, Boster, Wuhan, China). The blots were washed with TBST (1% Tween in Tris-buffered saline) on a shaker. Finally, the polyvinylidene fluoride membrane was incubated with HRP-conjugated goat anti-rabbit (H+L) cross-absorbed secondary antibody (1:10000, SA00001-2, Thermo Fisher Scientific, Waltham, MA USA) for 1 h, washed with TBST, and visualized with enhanced chemiluminescence reagent (Thermo Fisher Scientific, Waltham, MA USA).

### LC-MS/MS Analysis, Label-Free Quantification, and Bioinformatics Analysis

#### Sample Preparation

Tissue proteins were extracted in a microcentrifuge tube with lysate buffer (4% sodium dodecyl sulfate, 100 mM dithiothreitol, and 100 mM Tris-HCl; pH 8.0), boiled for 5 min, sonicated, and boiled again for 5 min. The sample was centrifuged at 20,000 *g* for 15 min and the supernatant was collected for protein quantification. Iodoacetamide, dithiothreitol, and detergent were added to the uric acid buffer, and the protein suspension was treated with trypsin (Promega, Madison, WI, USA) at a 50:1 ratio and incubated at 37°C overnight. Peptides collected after centrifugation at 12,000 *g* for 15 min were desalted with C18 Stage Tip for further LC-MS analysis (Easy 1200NLC, Thermo Fisher Scientific, Waltham, MA USA). Dithiothreitol, iodoacetamide, ammonium bicarbonate, and sodium carbonate were obtained from Sigma-Aldrich, St. Louis, MO, USA. Sodium dodecyl sulfate and urea were purchased from Bio-Rad, Hercules, CA, USA. Acetonitrile for nano-LC-MS/MS was obtained from J. T. Baker (Phillips, NJ, USA).

#### LC-MS/MS Analysis

Each sample was chromatographically separated following the manufacturer's instructions (Easy-nLC1200, Thermo Scientific). The column was first balanced with 95% phase A reagent (1% formic acid aqueous solution), and the samples were placed in the TRAP column and separated using gradient chromatography (300 nL/min). The peptides were separated using a linear gradient in buffer B (0.1% formic acid and 95% acetonitrile). Subsequently, they were separated and analyzed by data-dependent acquisition mass spectrometry using a Q-Exactive Plus mass spectrometer (Thermo Scientific).

#### Bioinformatics Analysis

Data were assessed using R statistical computing software, Perseus software (49), and Microsoft Excel 2020. Based on the statistical language R25, hierarchical clustering analysis was shown as a heatmap. The Euclidean distance with agglomeration method was used. Information was obtained from Gene Ontology (GO) (50), Kyoto Encyclopedia of Genes and Genomes (51), and UniProt KB/Swiss-Prot (52). Kyoto Encyclopedia of Genes and Genomes enrichment and GO analyses were performed along with Fisher's exact test, and false discovery rate correction was also performed with multiple testing. Three categories were grouped in GO terms: molecular functions, biological processes, and cellular components (50). Protein-protein interaction relationships were established using Cytoscape software in the STRING database (53).

### Immunofluorescence Analysis

Both NeuN and two key proteins related to the selected pathway were detected using a tissue immunofluorescence staining method described previously (54). Brain sections were fixed with 4% formalin and embedded in paraffin. Immunofluorescence and double immunofluorescence staining were performed by incubating deparaffinized sections with anti-NeuN monoclonal antibody (1:500, ab177487; Abcam, Waltham, MA USA), anti-clathrin polyclonal antibody (1:500, DF13215, Affinity, Cincinnati, OH, USA), and/or anti-EEA1 rabbit polyclonal antibody (1:500, 2411S, Cell Signaling Technology, Massachusetts, USA). All sections were incubated overnight with primary antibodies at 4°C and then incubated with rhodamine-labeled goat anti-rabbit IgG and goat anti-mouse IgG (1:500, ZF-0316, ZF-0313, Beijing Zhongshan Jinqiao Biological Co., Ltd., China). Nuclei were counterstained with 4′,6-diamidino-2-phenylindole. Immunofluorescence-labeled slides were imaged using a fluorescence scanning microscope (P250 flash, 3D HISTECH, Hungary) and a digital camera microscope (MoticBA410, Macaudi Industrial Group Co., Ltd. China). Immunofluorescence morphological images were set with the same image background to achieve the most objective and realistic experimental images.

### Screening of Key Proteins and Western Blot Analysis of Selected Proteins

Equal quantities (20 μg) of samples from different groups were separated using 10% sodium dodecyl sulfate-polyacrylamide gel electrophoresis. The protein was transferred onto a PVDF membrane (Millipore, Billerica, Massachusetts, USA), and the membrane was blocked using 5% bovine serum albumin for 2 h at 18–22°C and then incubated overnight with primary antibodies. Subsequent experimental procedures and reagents used were the same as those described above. The antibodies used in the study were anti-CHMP1a antibody (1:2000, ab167412, Abcam, Cambridge, MA, USA); anti-HSPA2 antibody (1:5000, ab108416, Abcam, Cambridge, MA, USA); anti-ARPC5 antibody (1:2,000, Novus Biologicals, Briarwood Avenue Centennial, USA); and anti-RABEP1 antibody (1:2,000, Novus Biologicals, Briarwood Avenue Centennial, USA).

### Statistical Approach

Most experimental data involved in this study were normally distributed (normality and lognormality tests were used). Data that were normally distributed are reported as mean ± standard deviation; one-way analysis of variance was used, and Tukey's test was applied to compare the difference between two groups. Kruskal–Wallis analysis was used for processing non-parametric data, data are expressed as median (interquartile range [IQR]), and Dunn's test was performed to compare the difference between two groups. All statistical tests were run using GraphPad Prism 9 (GraphPad, La Jolla, CA, USA). *P* < 0.05 were considered statistically significant. According to the proteomic analysis, label-free methods in Max Quant software were used for unlabeled quantitative calculation of proteomic data. In the process of significant difference analysis of quantitative results, at least two non-null values from three repeated experiments in the sample were screened for statistical analysis. A fold change of the proteins >1.5 times is considered statistically significant.

Adobe Illustrator CS5 (Adobe, San Jose, CA, USA) and Adobe Photoshop CS5 (Adobe, San Jose, CA, USA) were used to complete the layout and related artwork of the figures.

## Results

### Overview of the Experiment

Figure 1 shows the experimental design and procedures conducted in this study to explore the effects of IHP and PHP in MCAO rats. The following three preconditioned groups were set up: 2 h/day pretreatment group, 6 h/day pretreatment group (to observe the impact of different lengths of time under hypobaric hypoxia in MCAO rats), and the persistent hypobaric hypoxia preconditioned MCAO group. A combination of two different scoring systems (Longa scoring system and mNSS scoring system) was used to intuitively evaluate the extent of brain injury in the different groups from a behavioral perspective. TTC staining and neuron-specific marker staining were used to comprehensively analyze the degree of neuron injury. Transmission electron microscopy results were used to supplement the results from a microscopic point of view. Western blot analysis of apoptosis- and inflammation-related proteins was conducted; the best pretreatment group was selected for proteomic screening, which enabled further exploration of the possible mechanism underlying the neuroprotective effect of HHP.

### Effects of Persistent and Intermittent HHP on Infarct Volume and Neurobehavioral Outcomes

**Figure 2:**
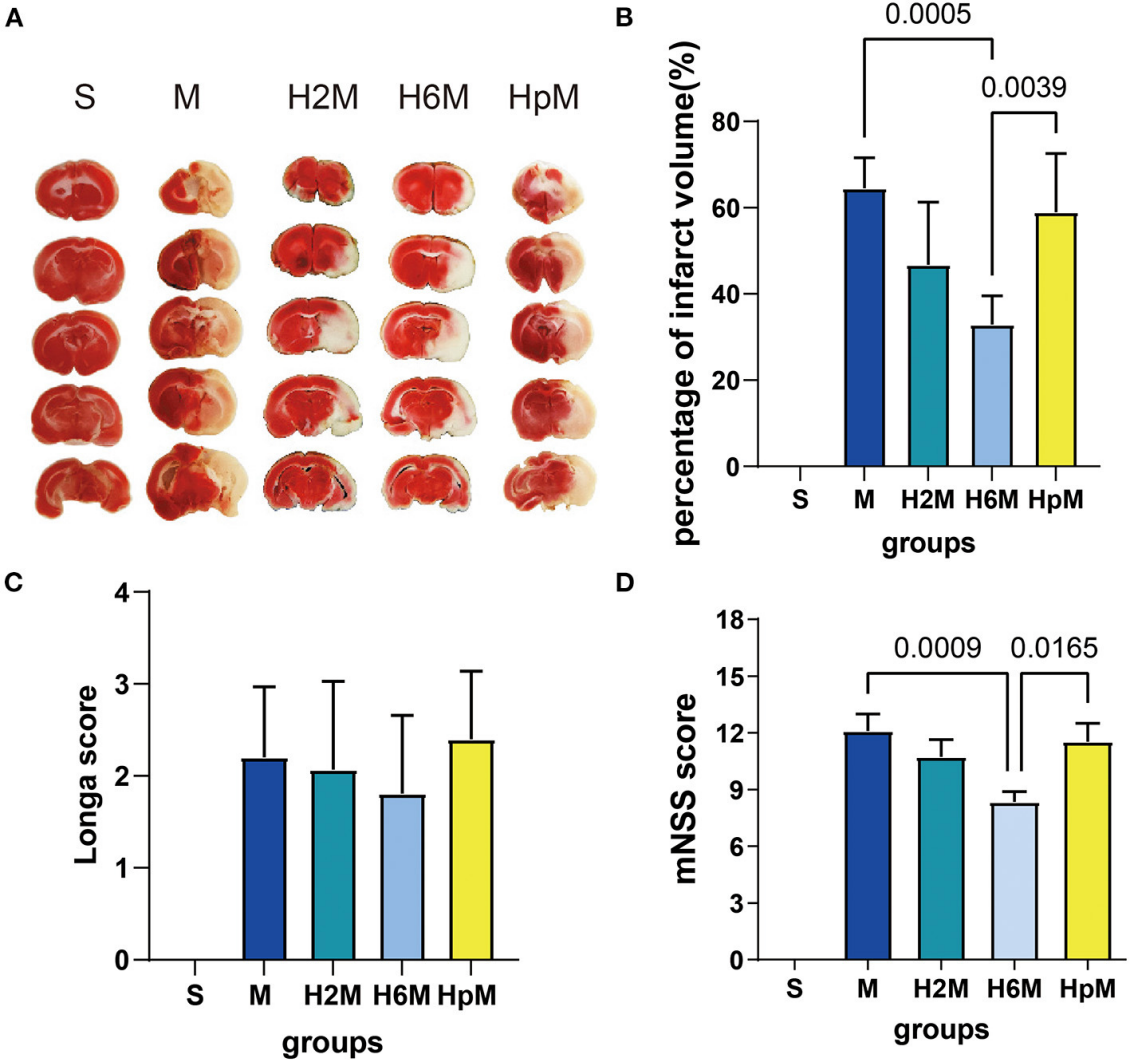
Effects of HHP on cerebral MCAO-induced ischemia. **(A)** 2,3,5-Triphenyl tetrazolium chloride (TTC) staining of the whole brain tissue. **(B)** Cerebral infarct volume calculation with edema correction 24 h after stroke. One-way ANOVA showed differences among the five groups, *F* = 33.89, *p* < 0.0001. Results are presented as mean ± SD (*n* = 5). **(C)** Longa score assessment 24 h after occlusion. One-way ANOVA showed differences among the five groups, *F* = 33.00, *p* < 0.0001. Results are presented as mean ± SD. S group (*n* = 20), M group (*n* = 20), H2M group (*n* = 15), H6M group (*n* = 26), HpM group (*n* = 15). **(D)** modified Neurological Severity Scores (mNSS) assessment 24 h after occlusion. One-way ANOVA showed differences among the five groups, *F* = 50.21, *p* < 0.0001. Results are presented as mean ± SD. S group (*n* = 20), M group (*n* = 20), H2M group (*n* = 15), H6M group (*n* = 26), HpM group (*n* = 15). ANOVA, analysis of variance; HHP, hypobaric hypoxia preconditioning; MCAO, middle cerebral artery occlusion.

The effects of HPC on the cortex of ischemic rats were assessed using 2,3,5-triphenyl tetrazolium chloride staining and neuropathological assessment (Figure 2 and Table 1). The infarct volume in the H6M group significantly decreased (32.91 ± 6.68, *p* = 0.0005) compared to that in the M group (64.50 ± 7.05), while the data in the other two preconditioned groups (H2M, 46.77 ± 14.53; HpM, 58.99 ± 13.56) showed no statistical difference (*p* > 0.05 for both) compared to the M group.

**Table 1:** Damage evaluation in rats after ischemia.

**Items (Mean ± SD)**	**S**	**M**	**H2M**	**H6M**	**HpM**	***F***	***p***
n (TTC)	5	5	5	5	5		
Infarct volume (%)	0.00 ± 0.00	64.50 ± 7.05 #	46.77 ± 14.53	32.91 ± 6.68 ##	58.99 ± 13.56	*F*_(4, 20)_ = 33.89	<0.0001
n (neurological score)	20	20	15	26	15		
Longa score	0.00 ± 0.00	2.20 ± 0.77	2.067 ± 0.96	1.81 ± 0.85	2.40 ± 0.74	*F*_(4, 91)_ = 33.00	<0.0001
mNSS score	0.00 ± 0.00^*^ ^**^ # ##	12.10 ± 3.97 #	10.73 ± 3.49	8.35 ± 2.79 ##	11.53 ± 3.80	*F*_(4, 91)_ = 50.21	<0.0001

*F, one-way analysis of variance test; H2M, 2 h/day intermittent hypobaric hypoxia preconditioned MCAO group; H6M, 6 h/day intermittent hypobaric hypoxia preconditioned MCAO group; HpM, persistent hypobaric hypoxia preconditioned MCAO group; M, middle cerebral artery occlusion (MCAO) group; mNSS, modified neurological severity score; n, number; p, p-value of ANOVA; S, sham; SD, standard deviation; TTC, 2,3,5-triphenyltetrazolium chloride*.

* *p < 0.05 vs. M group*,

** *p < 0.05 vs. H2M group*,

# *p < 0.05 vs. H6M group*,

## *p < 0.05 vs. HpM group*.

The mNSS score in the M group (12.10 ± 3.97) was higher than that in the S group, and it improved in the H6M group (8.35 ± 2.79, *p* = 0.0009) compared to the metric in the M group; the score in the HpM group (11.53 ± 3.80, *p* = 0.0165) was higher than that in the H6M group. For the Longa scoring system, there were no significant differences in the three preconditioned groups (*p* > 0.05 for all) compared to the MCAO group without HHP exposure.

### Ultrastructure of Vascular Endothelial Cells in the Peri-Infarct Region

**Figure 3:**
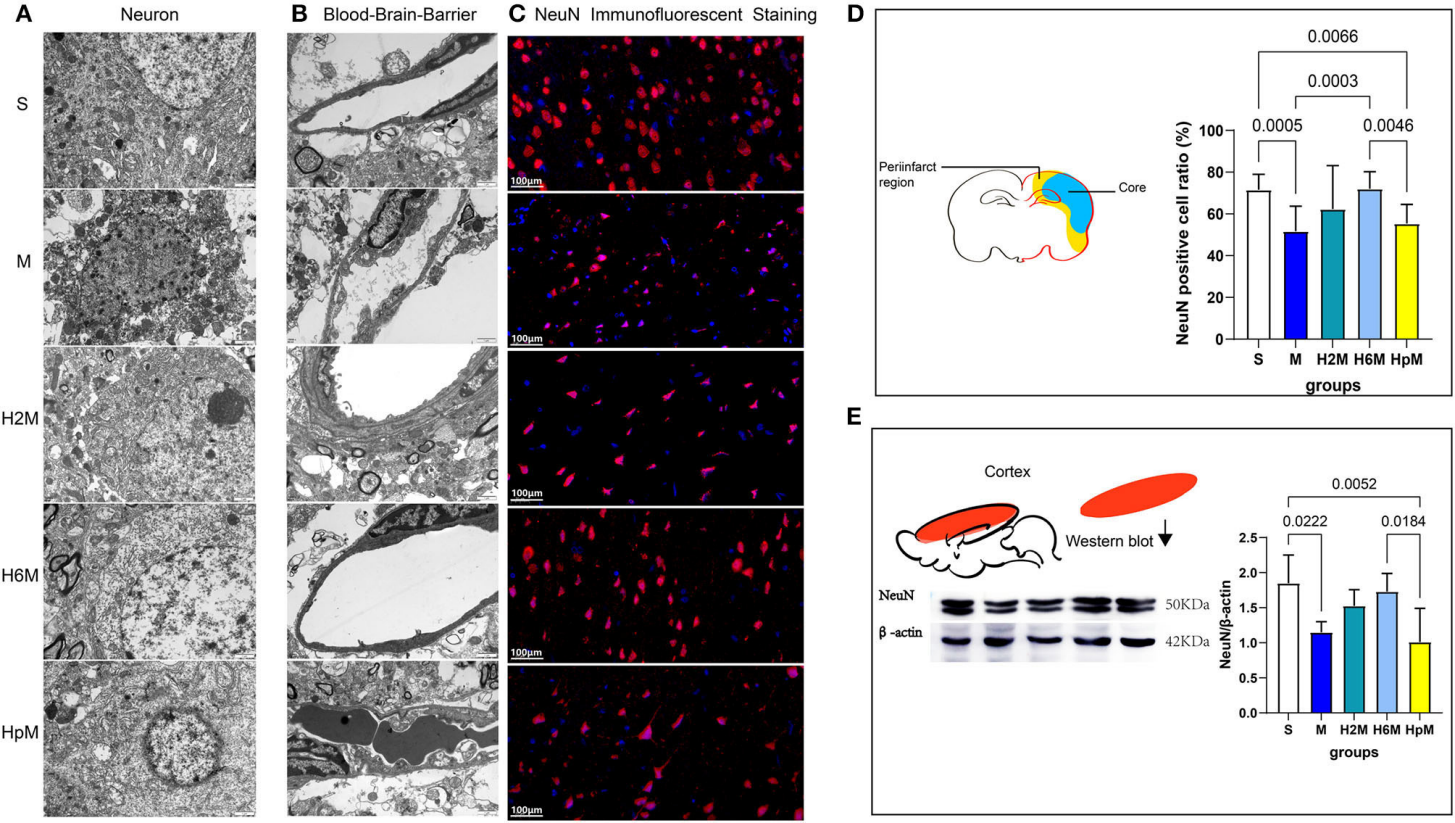
Ultrastructural changes of neurons, blood-brain barrier in the periinfarct zone, and assessment of NeuN in the damaged brain tissue. **(A)** Transmission electron microscopic observation of neurons (15,000 ×, 80 kV, *n* = 2). **(B)** Transmission electron microscopic observation of blood-brain barrier, vascular endothelial cells mainly (15,000 × , 80 kV, *n* = 2). **(C)** NeuN immunofluorescence staining in the periinfarct tissue (40×). **(D)** The diagram shows the periinfarct region and necrotic region (Core) of the MCAO rat's brain section and the NeuN positive cell ratio (%) [NeuN positive cell ratio (%) = (NeuN positive cell number/DAPI positive cell number)*100%] in the periinfarct. One-way ANOVA showed differences among the five groups, *F* = 8.08, *p* < 0.0001. Results are presented as mean ± SD (*n* = 5). ANOVA, analysis of variance; MCAO, middle cerebral artery occlusion; NeuN, neuron-specific nuclear protein. **(E)** Western blotting shows the results of NeuN in the damaged cerebral cortex. Statistical results of NeuN western blotting and mean gray value analysis of damaged cerebral cortex. One-way ANOVA showed differences among the five groups, *F* = 6.12, *p* = 0.0022. Results are presented as mean ± SD (*n* = 5).

Neurons and the blood-brain barrier in the peri-infarct tissue were observed using a transmission electron microscope (Figures 3A,B). The structure of cortical neurons and the blood-brain barrier in the S group was intact with no obvious abnormalities. The peri-infarct tissue in the M group had dissolved and cavitated, i.e., the perivascular space had widened; a large number of necrotic neurons with discontinuous cell membranes was observed; cytoplasmic contents were lost; most of the rough endoplasmic reticulum was dilated and cystic, and a large number of secondary lysosomes was observed in the cytoplasm. The nucleus of the vascular endothelial cells formed an irregular polygon, and organelles such as mitochondria, rough endoplasmic reticula, and ribosomes were observed in the cytoplasm. We recorded different degrees of cavitation in the four groups (M, H2M, H6M, and HpM) that underwent MCAO with the M group being the most severe. The mitochondrial structure was complete and clear; although most groups revealed signs of mild swelling to different degrees, the HpM and M groups were the most severely affected. Moreover, the perivascular space had widened to various degrees. In conclusion, the best overall structure of the ischemic peri-infarct tissue was observed in the H6M group and H2M group.

### Analysis of the Remaining Neurons in the Ischemic Peri-Infarct Tissue and NeuN Expression in the Damaged Cortex

Immunofluorescence staining for NeuN (Figure 3C) was conducted in five experimental animals from each group, at the same respective layers of the ischemic peri-infarct tissue (Figure 3D), under a 40 × microscope visual field. Three fields adjacent to the motor area were randomly selected for NeuN-positive cell ratio assessment (Figure 3D and Table 2). The NeuN-positive cell ratio (%) = (number of NeuN-positive cell / number of DAPI-positive cell)^*^100. The mean NeuN-positive cell ratio was 71.51 ± 7.44% in the S group, whereas it decreased sharply after stroke in the M group (51.74 ± 12.01%, *p* = 0.0005). This ratio increased in the H6M group (72.05 ± 8.20, *p* = 0.0003) compared to that in the M group. No significant difference was recorded in the residual neuron ratio between the S and H6M groups (*p* > 0.05). Furthermore, in the H6M group, neurons in the peri-infarct tissue were disorganized, and the cell morphology was altered compared to that in the S group. The mean NeuN-positive cell ratio (%) in the HpM (55.39 ± 9.15) decreased compared to that in the S (*p* = 0.0066) and H6M groups (*p* = 0.0046).

**Table 2:** NeuN mean gray value analyzed according to western blotting results and positive cell ratio based on immunofluorescence.

**NeuN (mean ± SD)**	**Groups**					***F***	***p***
	**S**	**M**	**H2M**	**H6M**	**HpM**		
n	5	5	5	5	5		
Western blotting analysis (NeuN/β-actin)	1.85 ± 0.40^*^##	1.15 ± 0.15	1.53 ± 0.23	1.73 ± 0.26 ##	1.01 ± 0.48	*F*_(4, 20)_ = 6.12	= 0.0022
NeuN positive cell ratio (%)	71.51 ± 7.44^*^##	51.74 ± 12.01 #	62.27 ± 20.87	72.05 ± 8.20 ##	55.39 ± 9.15	*F*_(4, 70)_ = 8.08	<0.0001

*F, one-way analysis of variance test; H2M, 2 h/day intermittent hypobaric hypoxia preconditioned MCAO group; H6M, 6 h/day intermittent hypobaric hypoxia preconditioned MCAO group; HpM, persistent hypobaric hypoxia preconditioned MCAO group; M, middle cerebral artery occlusion (MCAO) group; NeuN, neuron-specific nuclear protein; p, p-value of ANOVA; S, sham; SD, standard deviation*.

* p < 0.05 vs. M group, ^**^p < 0.05 vs. H2M group,

# *p < 0.05 vs. H6M group*,

## *p < 0.05 vs. HpM group*.

The protein expression of NeuN in the cortex varied among the five groups (Figure 3E and Table 2). The relative expression of NeuN in the M (1.15 ± 0.15) and HpM groups (1.01 ± 0.48) was lower (*p* < 0.05 for both) than that in the S group (1.85 ± 0.40). This value displayed an increasing trend in the H2M (1.53 ± 0.23) and H6M (1.73 ± 0.26) preconditioned groups, with no significant difference (*p* > 0.05 for both) as compared to the value in the M group.

### Expression of IL-6, Caspase-3, and Cleaved-Caspase-3 in Different Groups

**Figure 4:**
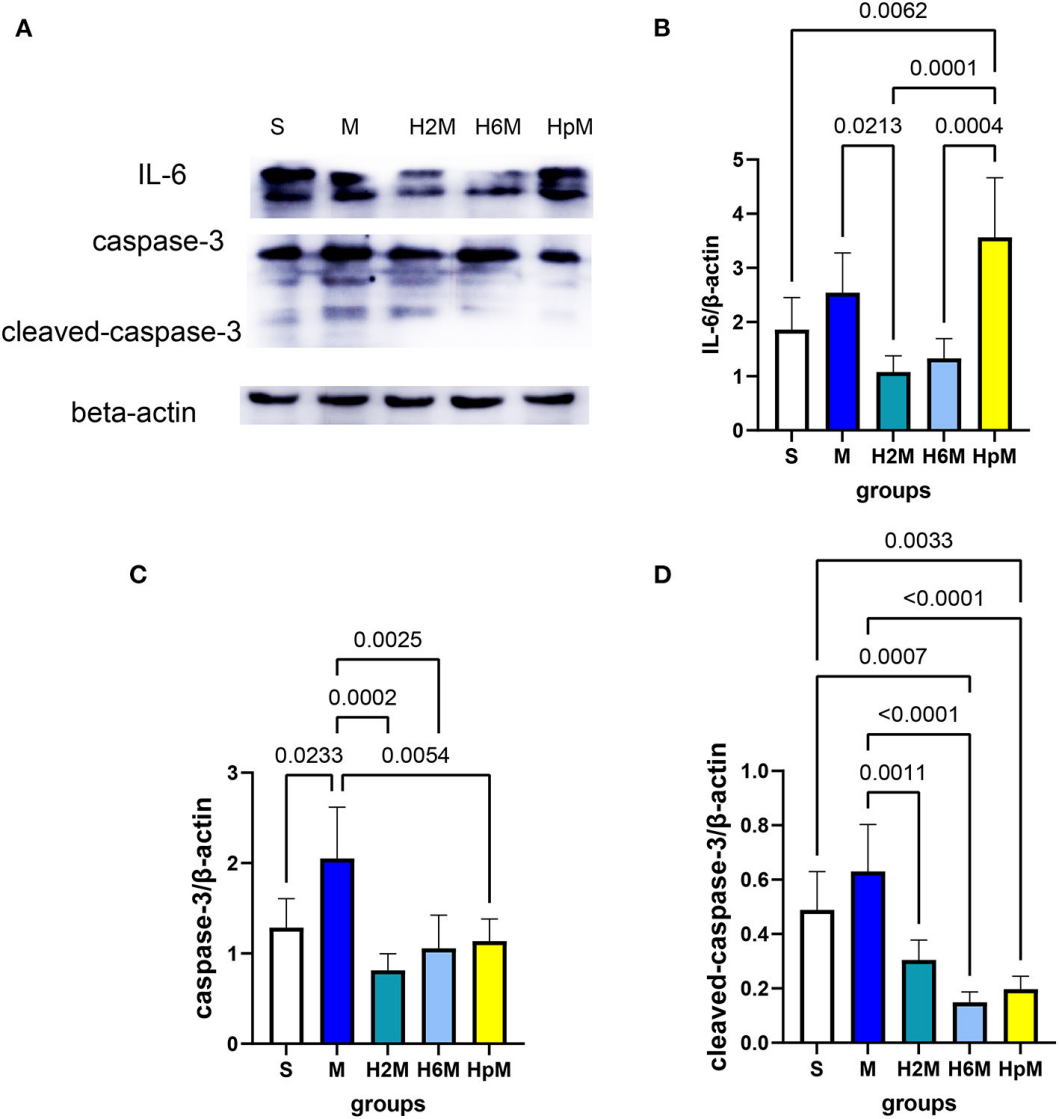
Relative expression of IL-6, caspase-3, and cleaved-caspase-3. **(A)** Immunoblot results of IL-6, caspase-3, and cleaved-caspase-3. **(B)** Analysis of IL-6 relative expression. One-way ANOVA showed differences among the five groups, *F* = 10.86, *p* < 0.0001. **(C)** Analysis of caspase-3 relative expression. One-way ANOVA showed differences among the five groups, *F* = 8.50, *p* = 0.0004. **(D)** Analysis of cleaved-caspase-3 relative expression. One-way ANOVA showed differences among the five groups, *F* = 17.36, *p* < 0.0001. ANOVA, analysis of variance; caspase-3, cysteinyl aspartate specific proteinase 3; IL-6, interleukin 6.

IL-6, caspase-3, and cleaved-caspase-3 levels in the cortex of the left cerebral hemisphere were evaluated using western blotting (Figure 4A and Table 3). The IL-6 level (Figure 4B) showed no difference in the M group (2.54 ± 0.73, *p* > 0.05) compared to that in the S group (1.86 ± 0.59). However, the IL-6 expression in the HpM group (3.57 ± 1.10) increased dramatically compared to that in the H2M group (1.08 ± 0.30, *p* = 0.0001) and H6M group (1.33 ± 0.36, *p* = 0.0004). In the 2 h preconditioned group, the IL-6 expression decreased (1.08 ± 0.30, *p* = 0.0213) compared to that in the M group. There were no differences between the M and H6M groups (1.33 ± 0.36, *p* > 0.05), and the IL-6 level in the HpM group was higher than that in the H2M and H6M groups (*p* < 0.05). The caspase-3 level (Figure 4C) increased in the M group (2.05 ± 0.57, *p* = 0.0233) compared to that in the S group (1.29 ± 0.32). This value decreased in the H2M (0.81 ± 0.18, *p* = 0.0002), H6M (1.06 ± 0.37, *p* = 0.0025), and HpM (1.14 ± 0.24, *p* = 0.0054) groups compared to that in the M group. The level of cleaved-caspase-3 (Figure 4D) was downregulated in the H2M (0.30 ± 0.07, *p* = 0.0011), H6M (0.15 ± 0.04, *p* < 0.0001), and HpM groups (0.20 ± 0.05, *p* < 0.0001) compared with that in the M group (0.63 ± 0.17). Those levels were markedly decreased in the H6M group (0.15 ± 0.04, *p* = 0.0007), and HpM group (0.20 ± 0.05, *p* = 0.0033) compared with those in the S group (0.49 ± 0.14).

**Table 3:** Mean gray value analyzed according to western blotting results of IL-6, caspase-3, and cleaved-caspase-3.

**Protein (mean ± SD)**	**Groups**					***F***	***p***
	**S**	**M**	**H2M**	**H6M**	**HpM**		
*n*	5	5	5	5	5		
Caspase-3	1.29 ± 0.32^*^	2.05 ± 0.57 ^**^ # ##	0.81 ± 0.18^*^	1.06 ± 0.37 ^*^	1.14 ± 0.24^*^	*F*_(4, 20)_ = 8.50	*=* 0.0004
Cleaved-caspase-3	0.49 ± 0.14# ##	0.63 ± 0.17 ^**^ # ##	0.30 ± 0.07^*^	0.15 ± 0.04 ^*^	0.20 ± 0.05^*^	*F*_(4, 20)_ = 17.36	<0.0001
IL-6	1.86 ± 0.59##	2.54 ± 0.73 ^**^	1.08 ± 0.30*##	1.33 ± 0.36 ##	3.57 ± 1.10^**^ ^#^	*F*_(4, 20)_ = 10.86	<0.0001

*Caspase-3, cysteinyl aspartate specific proteinase 3; F, one-way analysis of variance test; H2M, 2 h/day intermittent hypobaric hypoxia preconditioned MCAO group; H6M, 6 h/day intermittent hypobaric hypoxia preconditioned MCAO group; HpM, persistent hypobaric hypoxia preconditioned MCAO group; IL-6, interleukin-6; M, middle cerebral artery occlusion (MCAO) group; p, p-value of ANOVA; S, sham; SD, standard deviation*.

* *p < 0.05 vs. M group*,

** *p < 0.05 vs. H2M group*,

# *p < 0.05 vs. H6M group*,

## *p < 0.05 vs. HpM group*.

### Label-Free Proteomic Analysis and Western Blotting Verification

**Figure 5:**
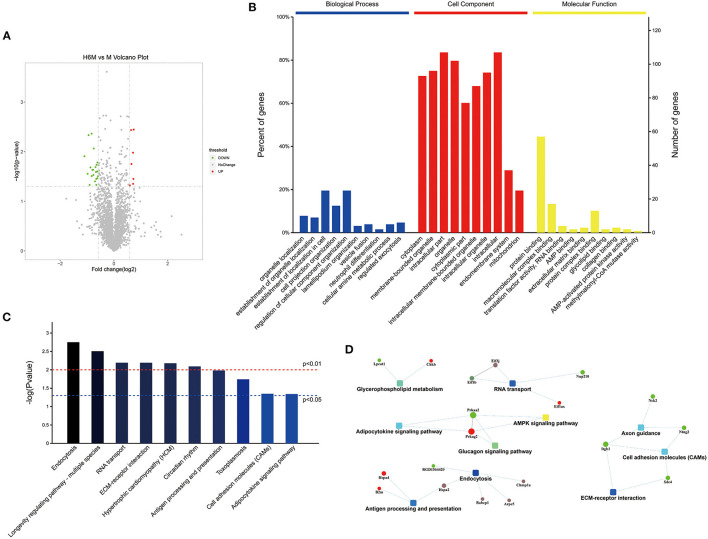
Functional proteomics and bioinformatics analysis between the M and H6M groups (*n* = 3). **(A)** Volcano map of differential protein analysis. The volcano map was drawn by the protein expression difference multiple between the two groups and the *p*-value obtained by the *t*-test. The abscissa is the multiple of the difference (logarithmic transformation with base 2), and the ordinate is the significant *p*-value of the difference (logarithmic transformation with base 10). Red represents the up-regulated protein, green represents the down-regulated protein, and gray represents the protein with no significant difference. **(B)** GO annotation and enrichment of differentially expressed proteins between the H6M and M groups. **(C)** Top 10 pathways of KEGG enrichment. **(D)** Network of the main potential proteins analyzed by MaxQuant. GO, Gene Ontology; KEGG, Kyoto Encyclopedia of Genes and Genomes.

To further explore the protective mechanism of HPC on cerebral ischemic injury, we selected the group with the most significant protective effect (H6M) and compared that to the infarction group without preconditioning (M) for proteomic analysis (Figure 5).

#### Screening of Differential Proteins and Pathways

A volcano plot was drawn as shown in Figure 5A. The fold change in protein expression and the *p*-value obtained by the *t*-test was used to assess the significant difference between the two groups. The abscissa was the multiple of the difference (logarithmic transformation based on 2), and the ordinate was the significant *p*-value of the difference (converted to - log10). The volcanic map shows the relative quantitative information of the H6M vs. M sample comparative histones as follows: the red dots indicate the significantly upregulated proteins, whereas the green dots indicate the significantly downregulated proteins. Significantly regulated proteins revealed by the *t*-test are presented as colored dots on the volcano map. Seven proteins were significantly upregulated, and 21 proteins were downregulated in the cortex of preconditioned MCAO rats.

GO data (Table 4) depict the enrichment of 28 different proteins that were significantly altered (*p* < 0.05) and interrelated among the 2,796 proteins identified (Supplementary Table 1); the change factor was set as 20 times higher or lower. The top enriched terms of location were intracellular, membrane-bounded organelle, and cytoplasm (Supplementary Tables 2–5). Regarding the cellular components (Figure 5B), compared to those in the M group, the significantly different proteins in the H6M group were cytoplasmic (70.99%), membrane-bounded organelle (73.28%), intracellular (81.68%), cytoplasmic (58.78%), organelle (77.86%), intracellular membrane-bounded organelle (66.41%), intracellular organelle (72.52%), endomembrane system (28.24%), and mitochondrion (19.08%). Most of these proteins are related to protein binding and molecular function and play a role in multiple biological processes, such as the establishment of cell localization (19.85%), regulation of cellular component organization (19.85%), and cell projection organization (12.21%). This process involves transport, catabolism (cellular processes), and translation. For all qualitative comparisons, as in the Kyoto Encyclopedia of Genes and Genomes pathway analysis, Fisher's exact test was used for calculating the meaningful enrichment of different proteins in each pathway (Figure 5C and Table 5) to determine the significantly influenced signal transduction pathways and metabolic changes. The top ten primarily affected signaling pathways were pathways associated with endocytosis, longevity regulation, RNA transport, ECM-receptor interaction, hypertrophic cardiomyopathy, circadian rhythm, antigen processing and presentation, toxoplasmosis, cell adhesion molecules, and adipocytokine signaling.

**Table 4:** GO annotation list of significantly different proteins (*n* = 3).

**No**.	**Protein name**	**Gene name**	**FoldChange**	**Log2FoldChange**	**Uniprot**
1	Rab GTPase-binding effector protein 1	*Rabep1*	1.5714246	0.65207305	O35550
2	Heat shock-related 70 kDa protein 2	*Hspa2*	1.640182505	0.713856354	P14659
3	Eukaryotic translation initiation factor 3 subunit J	*Eif3j*	1.507008193	0.59168726	A0JPM9
4	Cytochrome b5	*Cyb5a*	1.636974587	0.711031925	P00173
5	Charged multivesicular body protein 1a	*Chmp1a*	1.665224105	0.735716347	D4AE79
6	Protein CDV3 homolog	*Cdv3*	1.556275153	0.638097155	Q5XIM5
7	Actin-related protein 2/3 complex subunit 5	*Arpc5*	1.6517446	0.723990628	Q4KLF8
8	Ubiquitin carboxyl-terminal hydrolase; Ubiquitin carboxyl-terminal hydrolase isozyme L3	*Uchl3*	0.63448544	−0.656341038	Q91Y78
9	UBX domain-containing protein 1	*Ubxn1*	0.629564758	−0.667573311	Q499N6
10	MTOR-associated protein	*Tldc1*	0.546431232	−0.87188815	D4A255
11	SPARC-like protein 1	*Sparcl1*	0.563493967	−0.827527931	P24054
12	26S proteasome non-ATPase regulatory subunit 9	*Psmd9*	0.585102926	−0.773237661	Q9WTV5
13	Protein OSCP1	*OSCP1*	0.563573484	−0.827324359	Q4QQS3
14	Nipsnap homolog 3B	*Nipsnap3b*	0.508148532	−0.976677836	Q5M949
15	Methylmalonyl-CoA mutase	*Mut*	0.64085441	−0.641931454	D3ZKG1
16	Mitochondrial pyruvate carrier 1	*Mpc1*	0.638965701	−0.646189605	P63031
17	U6 snRNA-associated Sm-like protein LSm2	*Lsm2*	0.653782363	−0.613117637	Q6MG66
18	Laminin subunit gamma 1	*Lamc1*	0.635544098	−0.653935863	F1MAA7
19	Haloacid dehalogenase-like hydrolase domain-containing 3	*Hdhd3*	0.534630161	−0.903386865	B2RYT7
20	Glutaredoxin 5	*Glrx5*	0.468295786	−1.094508038	D4ADD7
21	Eukaryotic translation initiation factor 1B	*Eif1b*	0.595102412	−0.74879013	B5DFN1
22	Protein DPCD	*Dpcd*	0.622655582	−0.683493729	Q6AYM4
23	Cleavage and polyadenylation specific factor 6	*Cpsf6*	0.614264905	−0.703067134	D3ZPL1
24	Cyclin-dependent kinase 18	*Cdk18*	0.573545021	−0.802021359	O35832
25	Armadillo repeat-containing protein 1	*Armc1*	0.663015107	−0.592886352	F7F379
26	Adenine phosphoribosyltransferase	*Aprt*	0.654134205	−0.612341439	P36972
27	Abhydrolase domain containing 3	*Abhd3*	0.589266221	−0.763008528	D4A3D4
28	Keratin, type II cytoskeletal 5	*Krt5*	0.520572725	−0.941828371	Q6P6Q2

*GO, Gene Ontology*.

**Table 5:** KEGG pathway annotation list (*n* = 3).

**No**.	**Pathway name**	**Pathway ID**	***p*** **-value**	***p*** **-value_adjusted**	**Genes**	**Count**	**Pop hit**
1	Endocytosis	rno04144	0.00178	0.103	*Rabep1*|1.57142459984; *Hspa2*|1.64018250472; *Chmp1a*|1.66522410456; *Arpc5*|1.65174460005; *RGD1564420*|0.05; *Rab11fip2*|0.05	6	290
2	Longevity regulating pathway - multiple species	rno04213	0.00312	0.103	*Hspa2*|1.64018250472; *Prkaa2*|0.05; *Prkag2*|20.0	3	65
3	RNA transport	rno03013	0.00641	0.103	*Eif3j*|1.50700819302; *Eif1b*|0.595102411912; *Nup210*|0.05; *Eif1ax*|20.0	4	165
4	ECM-receptor interaction	rno04512	0.00642	0.103	*Lamc1*|0.635544097867; *Sdc4*|0.05; *Itgb1*|0.05	3	84
5	HCM	rno05410	0.00663	0.103	*Prkaa2*|0.05; *Itgb1*|0.05; *Prkag2*|20.0	3	85
6	Circadian rhythm	rno04710	0.0081	0.105	*Prkaa2*|0.05; *Prkag2*|20.0	2	30
7	Antigen processing and presentation	rno04612	0.0104	0.116	*Hspa2|*1.64018250472; *Hspa4*|20.0; *B2m*|20.0	3	100
8	Toxoplasmosis	rno05145	0.0181	0.176	*Hspa2*|1.64018250472; *Lamc1*|0.635544097867; *Itgb1*|0.05	3	123
9	CAMs	rno04514	0.0447	0.335	*Sdc4*|0.05; *Ntng2*|0.05; *Itgb1*|0.05	3	175
10	Adipocytokine signaling pathway	rno04920	0.0455	0.335	*Prkaa2*|0.05; *Prkag2*|20.0	2	75

*CAM, cell adhesion molecule; HCM, Hypertrophic cardiomyopathy; KEGG, Kyoto Encyclopedia of Genes and Genomes*.

The protein-protein interaction network (Figure 5D) was analyzed among meaningful pathways, revealing their functional interrelations. Hspa2 was associated with both endocytosis and antigen processing and presentation. Prkaa2 and Prkag2 were involved in the functioning of three pathways (adipocytokine signaling pathway, glucagon signaling pathway, and AMPK signaling pathway). The highly aggregated proteins related to endocytosis were as follows: Chmp1a, Rabep1, Arpc5, Hspa2, RGD1564420, and Rab11fip2. Additionally, among the top 10 pathways identified (Table 5), several were highly related to cardiac function (hypertrophic cardiomyopathy, circadian rhythm) and immunology (ECM-receptor interaction, cell adhesion molecules).

#### Protein Verification

**Figure 6:**
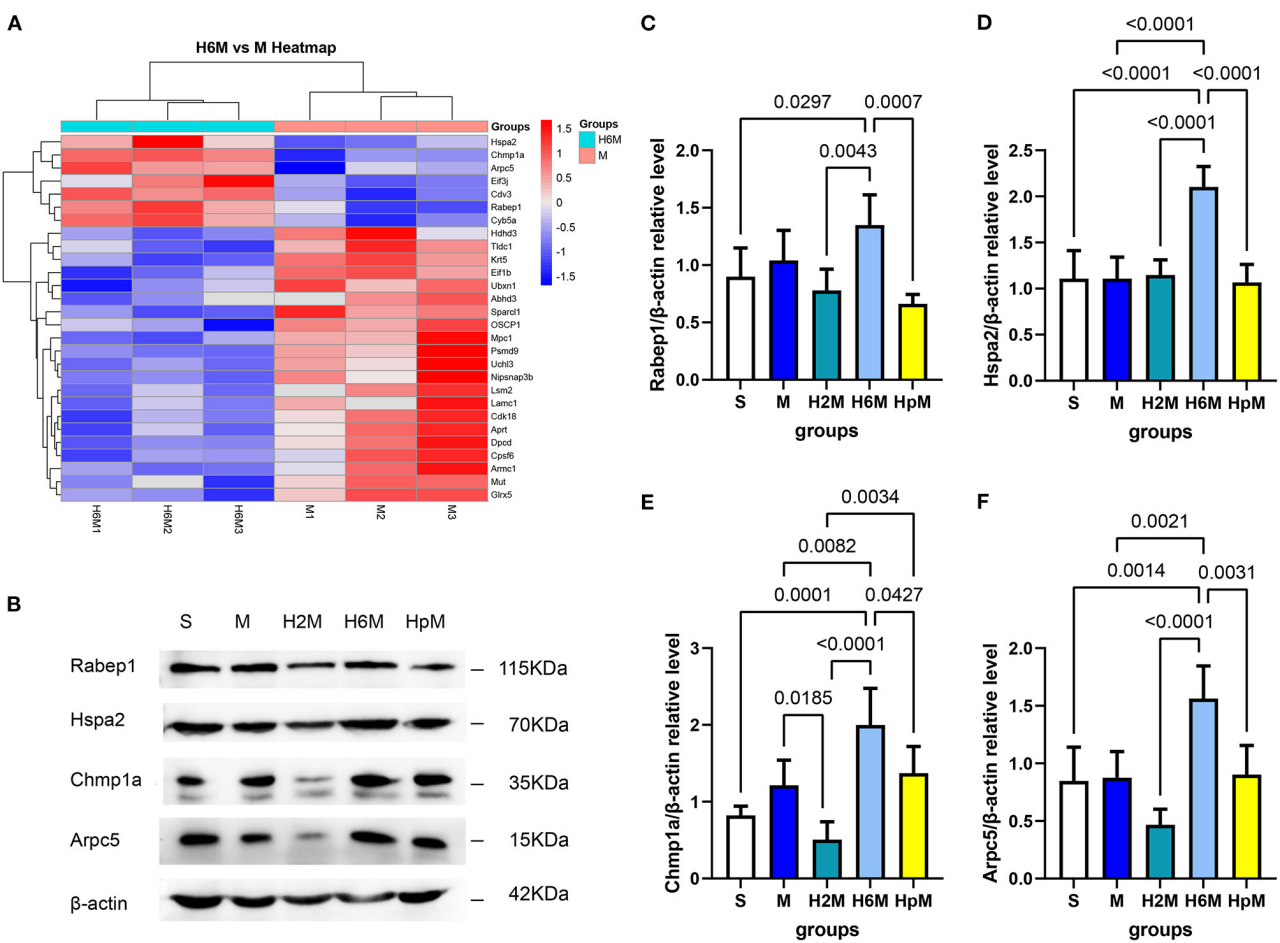
Cluster analysis of significantly different protein expressions and western blot analysis of selected key proteins. **(A)** Cluster analysis (H6M vs. M). Red represents up-regulation and blue represents down-regulation. **(B)** Western blotting results of four differentially expressed endocytosis-related proteins. **(C)** A western blotting gray value analysis of Rabep1. One-way ANOVA showed differences among the five groups, *F* = 7.34, *p* = 0.0008. Results are presented as mean ± SD (*n* = 5). **(D)** A western blotting gray value analysis of Hspa2. One-way ANOVA showed differences among the five groups, *F* = 19.29, *p* < 0.0001. Results are presented as mean ± SD (*n* = 5). **(E)** A western blotting gray value analysis of Chmp1a. One-way ANOVA showed differences among the five groups, *F* = 15.43, *p* < 0.0001. Results are presented as mean ± SD (*n* = 5). **(F)** A western blotting gray value analysis of Arpc5. One-way ANOVA showed differences among the five groups, *F* = 13.04, *p* < 0.0001. Results are presented as mean ± SD (*n* = 5). ANOVA, analysis of variance.

Based on the proteomic results mentioned above, the relative expression of key proteins was calculated (Figure 6 and Supplementary Table 6). According to the heatmap results, the expression of these proteins was highly related to CDE (Figure 6A). No significant change (*p* > 0.05) was recorded in any of the four proteins (Chmp1a, Rabep1, Arpc5, Hspa2) in the M group, compared to those in the S group. Furthermore, the expression of Rabep1 in the H6M group (1.35 ± 0.26) was higher than that in the S group (0.90 ± 0.25, *p* = 0.0297), H2M group (0.78 ± 0.19, *p* = 0.0043), and HpM group (0.66 ± 0.08, *p* = 0.0007). Expression of Hspa2 in the H6M group (2.10 ± 0.22) was higher than that in the S group (1.11 ± 0.30, *p* < 0.0001), M group (1.11 ± 0.23, *p* < 0.0001), H2M group (1.15 ± 0.16, *p* < 0.0001), and HpM group (1.07 ± 0.19, *p* < 0.0001). The relative expression of Chmp1a in the H6M group (2.00 ± 0.48) was higher than that in the S (0.82 ± 0.12, *p* = 0.0001), M (1.21 ± 0.33, *p* = 0.0082), H2M (0.51 ± 0.23, *p* < 0.0001), and HpM groups (1.37 ± 0.35, *p* = 0.0427). The relative expression of Arpc5 in the H6M group (1.56 ± 0.28) was higher than that in the S (0.85 ± 0.29, *p* = 0.0014), M (0.88 ± 0.23, *p* = 0.0021), H2M (0.47 ± 0.14, *p* < 0.0001), and HpM groups (0.90 ± 0.25, *p* = 0.0031). The expression of Hspa2, Chmp1a, and Arpc5 in the H6M group was significantly higher than that in the M group. This is partly consistent with previous proteomic results (Figure 6A). Although no statistical difference was recorded (*p* > 0.05) in Rabep1 expression between the H6M and M groups, an increasing trend was observed.

### Morphological Verification

**Figure 7:**
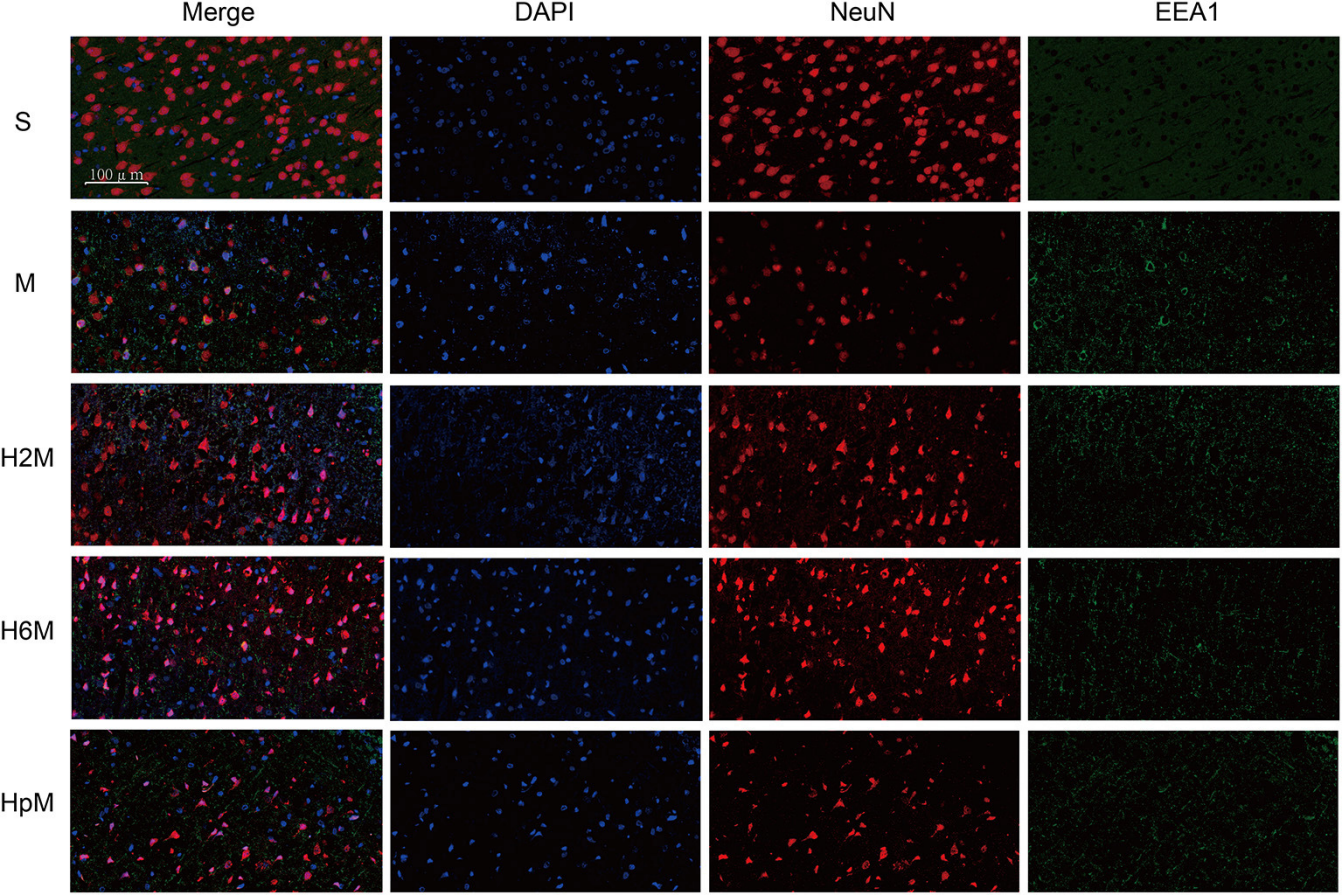
EEA1 and NeuN immunofluorescence double labeling. Neurons in the periinfarct region were co-stained with EEA1 positive staining cells after the MCAO procedure. The remaining neurons in each group showed positive staining of EEA1 in different degrees. Neurons in the four MCAO groups showed EEA1 positive staining, while it was not observed in the sham group. EEA1, Early endosome antigen 1; NeuN, neuron-specific nuclear protein.

**Figure 8:**
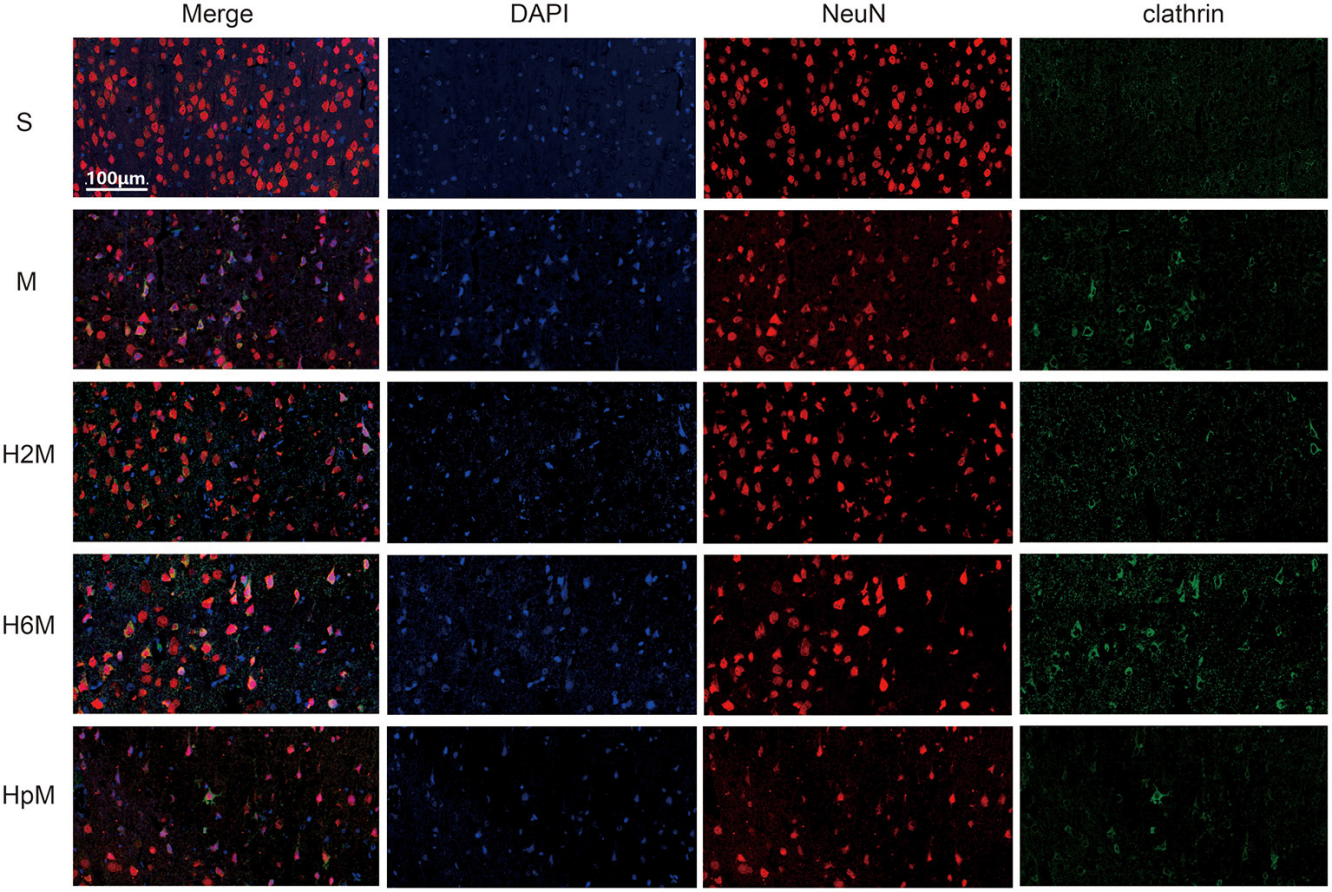
Clathrin and NeuN immunofluorescence double labeling. Neurons in the periinfarct region were co-stained with clathrin-positive cells after the MCAO procedure. The remaining neurons in each group showed positive staining of clathrin in different degrees. Neurons in the periinfarct region of rats after MCAO surgery showed partial clathrin-positive staining, while no clathrin-positive cells were observed in other cell types. NeuN, neuron-specific nuclear protein.

Early endosome antigen 1 (EEA1) and clathrin are key markers of CDE. Figures 7, 8 show the location of these two proteins; they were mainly present in the residual neurons (co-stained with NeuN in the peri-infarct tissue near the motor cortex). The EEA1-positive ratio in each group was calculated (Supplementary Figure 1A and Supplementary Table 7). The EEA1-positive cell ratio (%) = (number of EEA1-positive cells/number of DAPI-positive cells)^*^100. The EEA1-positive cell ratio in the M group was 20.34% (IQR: 15.56–33.85%); it markedly increased (*p* < 0.0001) compared to that in the S group [0.77&% (IQR: 0.00–1.42%)], while it decreased in the H2M [2.13% (IQR: 0.00–10.83%); *p* = 0.0021] and H6M groups [1.03% (IQR: 0.00–5.63%); *p* = 0.0005], compared to that in the M group. The clathrin-positive ratio was calculated (Supplementary Figure 1B and Supplementary Table 7). The clathrin-positive cell ratio (%) = (number of clathrin-positive cells/number of DAPI-positive cells)^*^100. In the S group, particularly in the cortex, clathrin-positive cells were scattered and mainly co-stained with NeuN-positive cells. The remaining NeuN-positive neurons in the peri-infarct tissue showed strong clathrin co-expression, with a higher positive ratio after MCAO [38.67% (IQR: 33.33–44.44%)], compared to that in the S group [10.48% (IQR: 5.50–21.65%); *p* = 0.0002]. Only the ratio in the H2M group decreased [22.41% (IQR: 17.02–30.68); *p* = 0.0139] compared to that in the M group.

## Discussion

Simulation of the plateau environment in hypobaric and hypoxic chambers is the optimum method to mimic the natural hypoxic environment. Based on previous research results, experimental conditions of 5,000 m (55) are relatively mild and rarely cause direct animal death.

Various animal behavioral scoring systems (56) exist to assess the extent of brain injury; among these, the Zea-Longa scoring system and mNSS scoring system are widely applied (57, 58). The Longa scoring system is simple, while the mNSS scoring system is more comprehensive. The neurological deficits in the ischemic rats, revealed by the two neurological scoring systems in our study, were not completely consistent. This phenomenon has not been described in other stroke studies. The mNSS neurological scoring system is more exhaustive than the Zea-Longa 5-level scoring system because of it includes more details. Conditioning for 6 h/day in a hypobaric hypoxia chamber for 10 days exhibited a significant neuroprotective phenomenon according to the estimation of both infarct volume and the behavioral scoring system, whereas the protective effect was unstable when the pre-treatment time was too short (2 h). Moreover, the total expression of NeuN in the 3 preconditioned groups did not exhibit statistically significant changes. However, the increasing trend evident in the data in comparison to the stroke model suggests that future studies involving larger cohorts and optimizing the duration of exposure for different groups are warranted.

IL-6 and caspase-3 are two important proteins involved in the inflammation and apoptosis after cerebral ischemia. IL-6 is a typical marker used to predict stroke prognosis. Its overexpression aggravates the inflammatory response of damaged tissue and affects the clinical effect of tissue plasminogen activator in the treatment of stroke (59). In this study, no striking changes were detected in rat brains 24 h after stroke, which is not in agreement with previous findings (59, 60). Two hours of preconditioning reduced the IL-6 protein level in the brain after infarction. Caspase-3 plays a pivotal role in cell apoptosis (61), particularly in neurons, owing to their high energy demand, with an increase in either caspase-3 or cleaved-caspase-3 being harmful to neuronal function (62). In this study, both IHP and PHP reduced the expression of caspase-3 and cleaved-caspase-3; HHP helped reduce apoptosis in ischemic brain tissue, which is in agreement with the results of other studies in which preconditioning inhibited apoptosis (63).

Compared with the irreparably damaged core necrotic area, the ischemic peri-infarct tissue possesses reversible neural function and is more valuable in the study of cerebral ischemic tolerance, which is also reflected in our experimental results. Immunofluorescence staining revealed that positive staining was mainly concentrated in the peri-infarct cortex. CDE may ameliorate the efficacy of some neuroprotective drugs (64), while many endogenous neuroprotective factors, such as vascular endothelial growth factor and brain-derived neurotrophic factor (65), may also exert neuroprotective effects through this pathway. The use of upregulated endocytosis in the ischemic peri-infarct tissue neurons after preconditioning could potentially improve the ischemic tolerance of brain tissue through endogenous neuroprotective pathways and exogenous drug intervention.

Endocytosis mediates an array of biological processes, such as receptor internalization, signal transduction, and nutrient absorption, all of which are crucial for cell survival and growth (40). In the nervous system, it also contributes markedly to neurotransmitter signaling and long-term inhibition of glutamate receptors (66). The mechanism mediating the brain-protective effect of hypoxic preconditioning may be related to endocytosis in neurons. This change is not directly related to clathrin expression but to the modulation of key molecules in this pathway, such as Arpc5, Chmp1a, and Hspa2. EEA1 is the most representative marker of early endosomes (67). Interestingly, we found that HHP decreased the expression of EEA1, which is an important marker of endocytosis There are two possibilities: (a) to avoid the over-activation of CDE, residual neurons downregulate key proteins to balance themselves; (b) substances in the early endosome are transferred to the late endosome, increasing the consumption of early endosome-related proteins.

**Figure 9:**
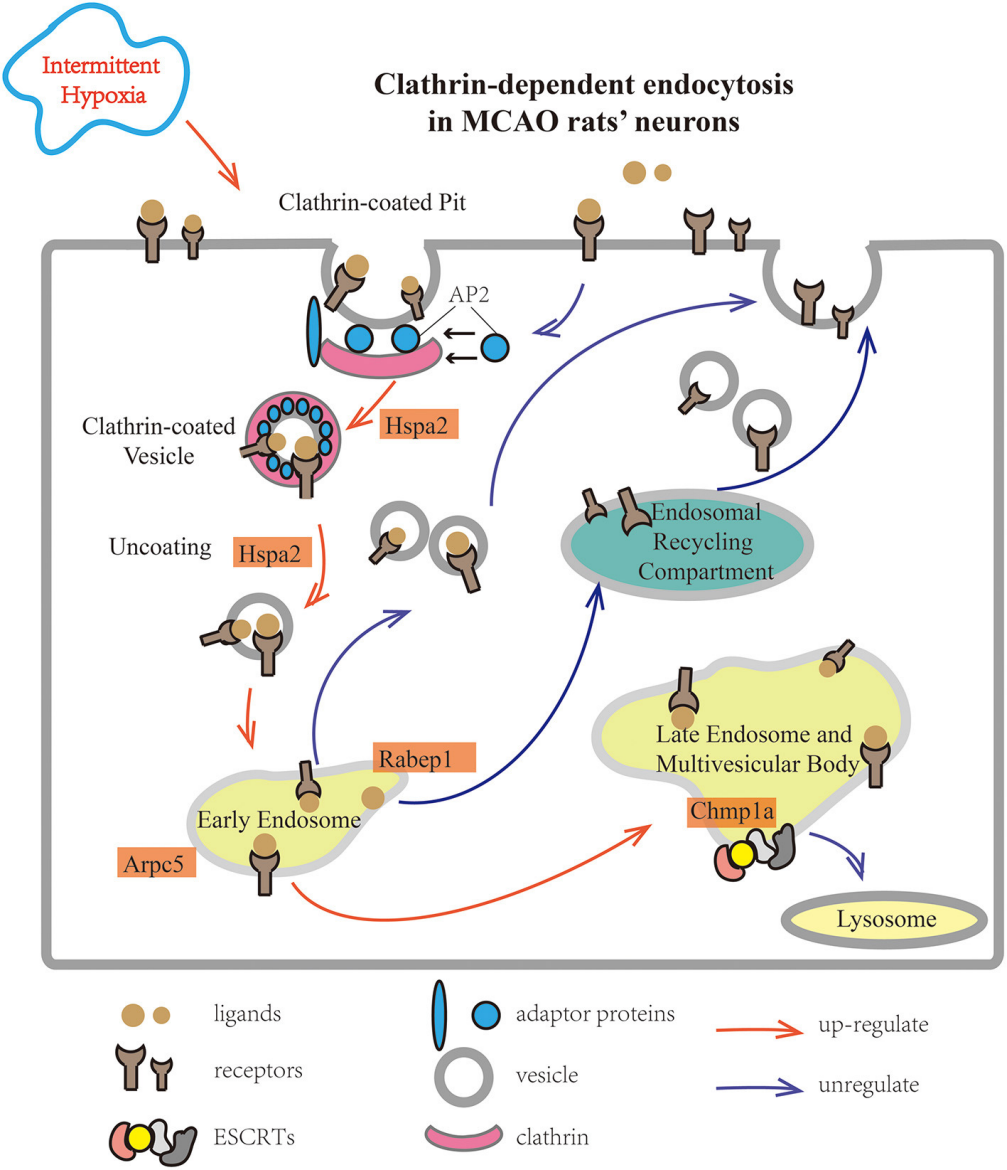
The main biological process changed in preconditioned MCAO rats' cortex based on KEGG pathways. After combining clathrin and adaptor proteins on the cytoplasmic side of the cell membrane, the signal receptors were collected on the surface of the cell membrane into clathrin-coated pits. Next, the pits were invaginated and pinched off the clathrin-surrounded membrane, the uncoated vesicle fused with the early endosome using Hspa2. This process included two factors, Arpc5 and Rabep1, which were up-regulated in the intermittent hypobaric hypoxia preconditioned group. In the case of CDE, the main outcomes were: (1) return to the cell membrane through the endosomal recycling compartment, and (2) entry into late endosomes and multivesicular bodies, and degradation by the lysosomal pathway. ESCRTs play a pivotal role during this process, and Chmp1a is essential for its function. KEGG, Kyoto Encyclopedia of Genes and Genomes; MCAO, middle cerebral artery occlusion.

Our data indicate that IHP exerts a better neuroprotective effect in rats with ischemic brain injury than persistent pre-treatment. IHP exhibited decreased infarct volume and modified neurological behavior after a stroke, the optimal duration being 6 h/day for 10 days. IHP activates key proteins related to CDE in the cortex, which is beneficial for medical treatment and ischemic tolerance. The ischemic neuron's CDE pathway is modulated by HHP (Figure 9), mainly by upregulating several key proteins in this pathway, promoting the entry of protective endogenous factors into the cells. Conversely, to avoid the over activation by this process, leading to cell damage, activation of CDE is not achieved through alterations in clathrin, rather by the changes in the expression of several proteins (including Rabep1, Hspa2, Chmp1a, Arpc5). Furthermore, activation of CDE in the central nervous system provides a new approach for targeting neuroprotective drugs to the neurons more effectively (68). This study has some limitations. The time grouping was not sufficiently detailed and lacked a clear time gradient. Moreover, because of the considerable differences between human and rat brains, further research is required to translate this basic research into clinical practice.

In conclusion, IHP exerted a neuroprotective effect in MCAO rats (improved outcomes, reduced cell apoptosis, and regulated the inflammatory response). The neuroprotective effect after 6 h/day of IHP was better than that observed after 2 h/day. PHP exhibited no protective effect in MCAO rats. Neurons in the peri-infarct tissue are well-regulated by endocytosis and may participate in the protective mechanism of HHP.

## Data Availability Statement

The datasets presented in this study can be found in online repositories. The names of the repository/repositories and accession number(s) can be found in the article/Supplementary Material.

## Ethics Statement

The animal study was reviewed and approved by Qinghai University Medical College Ethical Committee.

## Author Contributions

YW: study design and data collection, original drafting, and statistical analysis. R-lG: project design, study supervision, manuscript revision, and acquisition of financial support. LH: technical assistance and data analysis. YL: manuscript revision and technical assistance. WJ and CL: methodology assistance. All authors contributed to the article and approved the submitted version.

## Funding

The authors declare that this study received funding from the Qinghai Plateau Medicine Clinical Research Center Project (2017-SF-l1), the National Natural Science Foundation of China (82072107), and the Qinghai Fundamental Scientific and Technological Research Plan (2018-ZJ-730 and 2019-SF-134).

## Conflict of Interest

The authors declare that the research was conducted in the absence of any commercial or financial relationships that could be construed as a potential conflict of interest.

## Publisher's Note

All claims expressed in this article are solely those of the authors and do not necessarily represent those of their affiliated organizations, or those of the publisher, the editors and the reviewers. Any product that may be evaluated in this article, or claim that may be made by its manufacturer, is not guaranteed or endorsed by the publisher.

## Abbreviations

CCA: common carotid artery
CDE: clathrin-dependent endocytosis
GO: Gene Ontology
HHP: hypobaric hypoxia preconditioning
HPC: hypoxic preconditioning
ICA: internal carotid artery
IHP: intermittent hypoxic preconditioning
MCAO: middle cerebral artery occlusion
mNSS: modified neurological severity score
NeuN: neuron-specific nuclear protein
PHP: persistent hypoxic preconditioning.

## References

[1] Rust R Gronnert L Gantner C Enzler A Mulders G Weber RZ. Nogo-A targeted therapy promotes vascular repair and functional recovery following stroke. Proc Natl Acad Sci USA. (2019) 116:14270–9. 10.1073/pnas.1905309116 31235580 PMC6628809

[2] Feigin VL Forouzanfar MH Krishnamurthi R Mensah GA Connor M Bennett DA. Global and regional burden of stroke during 1990-2010: findings from the Global Burden of Disease Study 2010. Lancet. (2014) 383:245–55. 10.1016/S0140-6736(13)61953-4 24449944 PMC4181600

[3] Hankey GJ. Secondary stroke prevention. Lancet Neurol. (2014) 13:178–94. 10.1016/S1474-4422(13)70255-2 24361114

[4] Yang B Li W Satani N Nghiem DM Xi X Aronowski J. Protective effects of autologous bone marrow mononuclear cells after administering t-PA in an embolic stroke model. Transl Stroke Res. (2018) 9:135–45. 10.1007/s12975-017-0563-1 28836238

[5] Liu S Feng X Jin R Li G. Tissue plasminogen activator-based nanothrombolysis for ischemic stroke. Expert Opin Drug Deliv. (2018) 15:173–84. 10.1080/17425247.2018.1384464 28944694 PMC5780255

[6] Malik P Anwar A Patel R Patel U. Expansion of the dimensions in the current management of acute ischemic stroke. J Neurol. (2020) 268:3185–202. 10.1007/s00415-020-09873-6 32436103

[7] Phipps MS Cronin CA. Management of acute ischemic stroke. BMJ. (2020) 368:l6983. 10.1136/bmj.l6983 32054610

[8] Venkat P Shen Y Chopp M Chen J. Cell-based and pharmacological neurorestorative therapies for ischemic stroke. Neuropharmacology. (2018) 134:310–22. 10.1016/j.neuropharm.2017.08.036 28867364 PMC5832535

[9] Diener HC Hankey GJ. Primary and secondary prevention of ischemic stroke and cerebral hemorrhage: JACC focus seminar. J Am Coll Cardiol. (2020) 75:1804–18. 10.1016/j.jacc.2019.12.072 32299593

[10] Murry CE Jennings RB Reimer KA. Preconditioning with ischemia: a delay of lethal cell injury in ischemic myocardium. Circulation. (1986) 74:1124–36. 10.1161/01.CIR.74.5.1124 3769170

[11] M Weih K Kallenberg A Bergk UD L Harms KDW Einhäupl KM. Attenuated stroke severity after prodromal TIA: a role for ischemic tolerance in the brain? Stroke. (1999) 30:1851–4. 10.1161/01.STR.30.9.1851 10471435

[12] Sommer C. Neuronal plasticity after ischemic preconditioning and TIA-like preconditioning ischemic periods. Acta Neuropathol. (2009) 117:511–23. 10.1007/s00401-008-0473-0 19084975

[13] Ramos-Araque ME Rodriguez C Vecino R Cortijo Garcia E de Lera Alfonso M Sanchez Barba M. The neuronal ischemic tolerance is conditioned by the Tp53 Arg72Pro polymorphism. Transl Stroke Res. (2019) 10:204–15. 10.1007/s12975-018-0631-1 29687302 PMC6421278

[14] Jinggui Gao Zhenxiu Qin Xiang Qu Shuang Wu Xiaoyun Xie Chengwei Liang. Endogenous neuroprotective mechanism of ATP2B1 in transcriptional regulation of ischemic preconditioning. Am J Transl Res. (2021) 13:1170–83. 33841647 PMC8014370

[15] Taraseviciene-Stewart L Kasahara Y Alger L Hirth P Mc Mahon G Waltenberger J. Inhibition of the VEGF receptor 2 combined with chronic hypoxia causes cell death-dependent pulmonary endothelial cell proliferation and severe pulmonary hypertension. FASEB J. (2001) 15:427–38. 10.1096/fj.00-0343com 11156958

[16] Villegas LR Kluck D Field C Oberley-Deegan RE Woods C Yeager ME. Superoxide dismutase mimetic, MnTE-2-PyP, attenuates chronic hypoxia-induced pulmonary hypertension, pulmonary vascular remodeling, and activation of the NALP3 inflammasome. Antioxid Redox Signal. (2013) 18:1753–64. 10.1089/ars.2012.4799 23240585 PMC3619229

[17] Py G Eydoux N Lambert K Chapot R Koulmann N Sanchez H. Role of hypoxia-induced anorexia and right ventricular hypertrophy on lactate transport and MCT expression in rat muscle. Metabolism. (2005) 54:634–44. 10.1016/j.metabol.2004.12.007 15877294

[18] Pena E Brito J El Alam S Siques P. Oxidative stress, kinase activity and inflammatory implications in right ventricular hypertrophy and heart failure under hypobaric hypoxia. Int J Mol Sci. (2020) 21:6421. 10.3390/ijms21176421 32899304 PMC7503689

[19] Liu W Pu L Deng B Xu H Wang Z Wang T. Intermittent hypobaric hypoxia causes deleterious effects on the reproductive system in female rats. Biomed Pharmacother. (2020) 130:110511. 10.1016/j.biopha.2020.110511 32679462

[20] Wilson MH Newman S Imray CH. The cerebral effects of ascent to high altitudes. Lancet Neurol. (2009) 8:175–91. 10.1016/S1474-4422(09)70014-6 19161909

[21] Ray K Dutta A Panjwani U Thakur L Anand JP Kumar S. Hypobaric hypoxia modulates brain biogenic amines and disturbs sleep architecture. Neurochem Int. (2011) 58:112–8. 10.1016/j.neuint.2010.11.003 21075155

[22] Yu L Chen Y Wang W Xiao Z Hong Y. Multi-vitamin B supplementation reverses hypoxia-induced tau hyperphosphorylation and improves memory function in adult mice. J Alzheimers Dis. (2016) 54:297–306. 10.3233/JAD-160329 27497480

[23] Kumar K Sharma S Vashishtha V Bhardwaj P Kumar A Barhwal K. Terminalia arjuna bark extract improves diuresis and attenuates acute hypobaric hypoxia induced cerebral vascular leakage. J Ethnopharmacol. (2016) 180:43–53. 10.1016/j.jep.2016.01.002 26771070

[24] Beer JMA Shender BS Chauvin D Dart TS Fischer J. Cognitive deterioration in moderate and severe hypobaric hypoxia conditions. Aerosp Med Hum Perform. (2017) 88:617–26. 10.3357/AMHP.4709.2017 28641678

[25] Hou Y Wang X Chen X Zhang J Ai X Liang Y. Establishment and evaluation of a simulated highaltitude hypoxic brain injury model in SD rats. Mol Med Rep. (2019) 19:2758–66. 10.3892/mmr.2019.9939 30720143 PMC6423628

[26] Mishra S Kumar G Chhabra A Sethy NK Jain N Meena RN. Cysteine becomes conditionally essential during hypobaric hypoxia and regulates adaptive neuro-physiological responses through CBS/H2S pathway. Biochim Biophys Acta Mol Basis Dis. (2020) 1866:165769. 10.1016/j.bbadis.2020.165769 32184133

[27] Viscor G Torrella JR Corral L Ricart A Javierre C Pages T. Physiological and biological responses to short-term intermittent hypobaric hypoxia exposure: from sports and mountain medicine to new biomedical applications. Front Physiol. (2018) 9:814. 10.3389/fphys.2018.00814 30038574 PMC6046402

[28] Coimbra-Costa D Garzon F Alva N Pinto TCC Aguado F Torrella JR. Intermittent hypobaric hypoxic preconditioning provides neuroprotection by increasing antioxidant activity, erythropoietin expression and preventing apoptosis and astrogliosis in the brain of adult rats exposed to acute severe hypoxia. Int J Mol Sci. (2021) 22:5272. 10.3390/ijms22105272 34067817 PMC8156215

[29] Dirnagl U Becker K Meisel A. Preconditioning and tolerance against cerebral ischaemia: from experimental strategies to clinical use. Lancet Neurol. (2009) 8:398–412. 10.1016/S1474-4422(09)70054-7 19296922 PMC2668955

[30] Li Y Yu P Chang SY Wu Q Yu P Xie C. Hypobaric hypoxia regulates brain iron homeostasis in rats. J Cell Biochem. (2017) 118:1596–605. 10.1002/jcb.25822 27925282

[31] Zhan L Lu X Xu W Sun W Xu E. Inhibition of MLKL-dependent necroptosis via downregulating interleukin-1R1 contributes to neuroprotection of hypoxic preconditioning in transient global cerebral ischemic rats. J Neuroinflammation. (2021) 18:97. 10.1186/s12974-021-02141-y 33879157 PMC8056617

[32] Saito Y Nakamura M Eguchi K Otsuki T. Mild hypobaric hypoxia enhances post-exercise vascular responses in young male runners. Front Physiol. (2019) 10:546. 10.3389/fphys.2019.00546 31178742 PMC6543008

[33] Aguilar M Gonzalez-Candia A Rodriguez J Carrasco-Pozo C Canas D Garcia-Herrera C. Mechanisms of cardiovascular protection associated with intermittent hypobaric hypoxia exposure in a rat model: role of oxidative stress. Int J Mol Sci. (2018) 19:20366. 10.3390/ijms19020366 29373484 PMC5855588

[34] Lin HJ Wang CT Niu KC Gao C Li Z Lin MT. Hypobaric hypoxia preconditioning attenuates acute lung injury during high-altitude exposure in rats via up-regulating heat-shock protein 70. Clin Sci (Lond). (2011) 121:223–31. 10.1042/CS20100596 21599636

[35] Shrivastava K Shukla D Bansal A Sairam M Banerjee PK Ilavazhagan G. Neuroprotective effect of cobalt chloride on hypobaric hypoxia-induced oxidative stress. Neurochem Int. (2008) 52:368–75. 10.1016/j.neuint.2007.07.005 17706837

[36] Zhao Y Zhou Y Ma X Liu X Zhao Y Liu X. DDAH-1 via HIF-1 target genes improves cerebral ischemic tolerance after hypoxic preconditioning and middle cerebral artery occlusion-reperfusion. Nitric Oxide. (2020) 95:17–28. 10.1016/j.niox.2019.12.004 31870966

[37] Wakai T Narasimhan P Sakata H Wang E Yoshioka H Kinouchi H. Hypoxic preconditioning enhances neural stem cell transplantation therapy after intracerebral hemorrhage in mice. J Cereb Blood Flow Metab. (2016) 36:2134–45. 10.1177/0271678X15613798 26661220 PMC5363661

[38] Sun J Wei ZZ Gu X Zhang JY Zhang Y Li J. Intranasal delivery of hypoxia-preconditioned bone marrow-derived mesenchymal stem cells enhanced regenerative effects after intracerebral hemorrhagic stroke in mice. Exp Neurol. (2015) 272:78–87. 10.1016/j.expneurol.2015.03.011 25797577

[39] Prabhakar NR Semenza GL. Adaptive and maladaptive cardiorespiratory responses to continuous and intermittent hypoxia mediated by hypoxia-inducible factors 1 and 2. Physiol Rev. (2012) 92:967–1003. 10.1152/physrev.00030.2011 22811423 PMC3893888

[40] Doherty GJ McMahon HT. Mechanisms of endocytosis. Annu Rev Biochem. (2009) 78:857–902. 10.1146/annurev.biochem.78.081307.110540 19317650

[41] McMahon HT Boucrot E. Molecular mechanism and physiological functions of clathrin-mediated endocytosis. Nat Rev Mol Cell Biol. (2011) 12:517–33. 10.1038/nrm3151 21779028

[42] Calvo AC Olivan S Manzano R Zaragoza P Aguilera J Osta R. Fragment C of tetanus toxin: new insights into its neuronal signaling pathway. Int J Mol Sci. (2012) 13:6883–901. 10.3390/ijms13066883 22837670 PMC3397502

[43] Tian D Guo Y Zhang D Gao Q Liu G Lin J. Shenzhi Jiannao formula ameliorates vascular dementia *in vivo* and *in vitro* by inhibition glutamate neurotoxicity via promoting clathrin-mediated endocytosis. Chin Med. (2021) 16:65. 10.1186/s13020-021-00477-4 34321050 PMC8317332

[44] McBride DW Zhang JH. Precision stroke animal models: the permanent MCAO model should be the primary model, not transient MCAO. Transl Stroke Res. (2017). 10.1007/s12975-017-0554-2 28718030 PMC5772000

[45] Chen J Sanberg PR Li LW Lu M Willing AE Sanchez-Ramos J. Intravenous administration of human umbilical cord blood reduces behavioral deficits after stroke in rats. Stroke. (2001) 32:2682–8. 10.1161/hs1101.098367 11692034

[46] Longa EZ Weinstein PR Carlson S Cummins R. Reversible middle cerebral artery occlusion without craniectomy in rats. Stroke. (1989) 20:84–91. 10.1161/01.STR.20.1.84 2643202

[47] Chen J Li Y Wang L Zhang Z Lu D Lu M. Therapeutic benefit of intravenous administration of bone marrow stromal cells after cerebral ischemia in rats. Stroke. (2001) 32:1005–11. 10.1161/01.STR.32.4.1005 11283404

[48] Schäbitz WR Weber J Takano K Sandage BW Locke KW Fisher M. The effects of prolonged treatment with citicoline in temporary experimental focal ischemia. J Neurol Sci. (1996) 138:21–5. 10.1016/0022-510X(95)00341-X 8791234

[49] Tyanova S Temu T Sinitcyn P Carlson A Hein MY Geiger T. The Perseus computational platform for comprehensive analysis of (prote)omics data. Nat Methods. (2016) 13:731–40. 10.1038/nmeth.3901 27348712

[50] Ashburner M Ball CA Blake JA Botstein D Butler H Cherry JM. Gene ontology: tool for the unification of biology. The Gene Ontology Consortium. Nat Genet. (2000) 25:25–9. 10.1038/75556 10802651 PMC3037419

[51] Kanehisa M Goto S Sato Y Furumichi M Tanabe M. KEGG for integration and interpretation of large-scale molecular data sets. Nucleic Acids Res. (2012) 40:D109–14. 10.1093/nar/gkr988 22080510 PMC3245020

[52] Boutet E Lieberherr D Tognolli M Schneider M Bansal P Bridge AJ. UniProtKB/swiss-prot, the manually annotated section of the UniProt knowledgebase: how to use the entry view. Methods Mol Biol. (2016) 1374:23–54. 10.1007/978-1-4939-3167-5_2 26519399

[53] Kohl M Wiese S Warscheid B. Cytoscape: software for visualization and analysis of biological networks. Methods Mol Biol. (2011) 696:291–303. 10.1007/978-1-60761-987-1_18 21063955

[54] Melani A Cipriani S Vannucchi MG Nosi D Donati C Bruni P. Selective adenosine A2a receptor antagonism reduces JNK activation in oligodendrocytes after cerebral ischaemia. Brain. (2009) 132:1480–95. 10.1093/brain/awp076 19359287

[55] Verges S Chacaroun S Godin-Ribuot D Baillieul S. Hypoxic conditioning as a new therapeutic modality. Front Pediatr. (2015) 3:58. 10.3389/fped.2015.00058 26157787 PMC4476260

[56] Zheng XW Yang WT Chen S Xu QQ Shan CS Zheng GQ. Neuroprotection of catalpol for experimental acute focal ischemic stroke: preclinical evidence and possible mechanisms of antioxidation, anti-inflammation, and antiapoptosis. Oxid Med Cell Longev. (2017) 2017:5058609. 10.1155/2017/5058609 28785376 PMC5530418

[57] Shi YH Zhang XL Ying PJ Wu ZQ Lin LL Chen W. Neuroprotective Effect of Astragaloside IV on Cerebral Ischemia/Reperfusion Injury Rats Through Sirt1/Mapt Pathway. Front Pharmacol. (2021) 12:639898. 10.3389/fphar.2021.639898 33841157 PMC8033022

[58] Zhang Y Cao M Wu Y Wang J Zheng J Liu N. Improvement in mitochondrial function underlies the effects of ANNAO tablets on attenuating cerebral ischemia-reperfusion injuries. J Ethnopharmacol. (2020) 246:112212. 10.1016/j.jep.2019.112212 31494200

[59] Armstead WM Hekierski H Pastor P Yarovoi S Higazi AA Cines DB. Release of IL-6 after stroke contributes to impaired cerebral autoregulation and hippocampal neuronal necrosis through NMDA receptor activation and upregulation of ET-1 and JNK. Transl Stroke Res. (2019) 10:104–11. 10.1007/s12975-018-0617-z 29476447

[60] Billah M Ridiandries A Allahwala UK Mudaliar H Dona A Hunyor S. Remote ischemic preconditioning induces cardioprotective autophagy and signals through the IL-6-dependent JAK-STAT pathway. Int J Mol Sci. (2020) 21:1692. 10.3390/ijms21051692 32121587 PMC7084188

[61] Porter AG Jänicke RU. Emerging roles of caspase-3 in apoptosis. Cell Death Differ. (1999) 6:99–104. 10.1038/sj.cdd.4400476 10200555

[62] Broughton BR Reutens DC Sobey CG. Apoptotic mechanisms after cerebral ischemia. Stroke. (2009) 40:e331–9. 10.1161/STROKEAHA.108.531632 19182083

[63] Guo M Ma X Feng Y Han S Dong Q Cui M. In chronic hypoxia, glucose availability and hypoxic severity dictate the balance between HIF-1 and HIF-2 in astrocytes. FASEB J. (2019) 33:11123–36. 10.1096/fj.201900402RR 31298941

[64] Zhao Y Xiong S Liu P Liu W Wang Q Liu Y. Polymeric nanoparticles-based brain delivery with improved therapeutic efficacy of ginkgolide B in Parkinson's disease. Int J Nanomedicine. (2020) 15:10453–67. 10.2147/IJN.S272831 33380795 PMC7769078

[65] Yan B Gao L Huang Y Wang X Lang X Yan F. Exosomes derived from BDNF-expressing 293T attenuate ischemic retinal injury *in vitro* and *in vivo*. Aging (Albany NY). (2020) 12:202245. 10.18632/aging.202245 33260157

[66] Diaz-Guerra M. Excitotoxicity-induced endocytosis as a potential target for stroke neuroprotection. Neural Regen Res. (2021) 16:300–1. 10.4103/1673-5374.290892 32859784 PMC7896228

[67] Wilson JM de Hoop M Zorzi N Toh BH Dotti CG Parton RG. EEA1, a tethering protein of the early sorting endosome, shows a polarized distribution in hippocampal neurons, epithelial cells, and fibroblasts. Mol Biol Cell. (2000) 11:2657–71. 10.1091/mbc.11.8.2657 10930461 PMC14947

[68] Vaslin A Naegele-Tollardo S Puyal J Clarke PG. Excitotoxicity-induced endocytosis mediates neuroprotection by TAT-peptide-linked JNK inhibitor. J Neurochem. (2011) 119:1243–52. 10.1111/j.1471-4159.2011.07535.x 22004371

