# Supplementary material for: Preconditioning With Intermittent Hypobaric Hypoxia Attenuates Stroke Damage and Modulates Endocytosis in Residual Neurons
Source: Front Neurol. 2021 Dec 15;12:750908. doi: 10.3389/fneur.2021.750908 (PMC8715922; doi:10.3389/fneur.2021.750908)
Supplement: Supplementary file 1 [file Data_Sheet_1.ZIP › supplementary data and figures/Supplemental Figure/Supplemental Figure Legend.docx]

Supplemental Figure 1. Positive cell ratio of EEA1 and clathrin in the ischemic penumbra

(A) EEA1 positive cell count and positive cell rate. Kruskal-Wallis test was used. Kruskal-Wallis statistic of EEA1 positive cell ratio = 27.89, *p* < 0.0001­. (B) Clathrin positive cell ratio. Kruskal-Wallis test was used. Kruskal-Wallis statistic of clathrin positive cell ratio = 25.62, p < 0.0001. Results are presented as median (interquartile range [IQR]) (n=5).

EEA1 positive cell ratio (%) =(EEA1 positive cell number/DAPI positive cell number)*100%］,［Clathrin positive cell ratio (%) =( Clathrin positive cell number/DAPI positive cell number)*100%］
