# Supplementary material for: Preconditioning With Intermittent Hypobaric Hypoxia Attenuates Stroke Damage and Modulates Endocytosis in Residual Neurons
Source: Front Neurol. 2021 Dec 15;12:750908. doi: 10.3389/fneur.2021.750908 (PMC8715922; doi:10.3389/fneur.2021.750908)
Supplement: Supplementary file 1 [file Data_Sheet_1.ZIP › supplementary data and figures/Supplemental Tables/Supplemental Table 6-7.docx]

**Supplemental Table 6-7**

Supplementary Table 6. Mean gray value analyzed according to western blotting results (proteomics verification)

| **Protein**  **(Mean ± SD)** | **Groups** | | | | | ***F*** | ***p*** |
| --- | --- | --- | --- | --- | --- | --- | --- |
|  | **S** | **M** | **H2M** | **H6M** | **HpM** |  |  |
| n | 5 | 5 | 5 | 5 | 5 |  |  |
| Rabep1 | 0.90 ± 0.25  # | 1.04 ± 0.26 | 0.78 ± 0.19  # | 1.35 ± 0.26  ## | 0.66 ± 0.08  # | *F* (4, 20) = 7.336 | = 0.0008 |
| Hspa2 | 1.11 ± 0.30  # | 1.11 ± 0.23  # | 1.15 ± 0.16  # | 2.10 ± 0.22  ##  *  ** | 1.07 ± 0.19  # | *F* (4, 20) = 19.29 | < 0.0001 |
| Chmp1a | 0.82 ± 0.12  # | 1.21 ± 0.33  #  ** | 0.51 ± 0.23  *  #  ## | 2.00 ± 0.48  *  **  ## | 1.37 ± 0.35  #  ** | *F* (4, 20) = 15.43 | < 0.0001 |
| Arpc5 | 0.85 ± 0.29  # | 0.88 ± 0.23  # | 0.47 ± 0.14  # | 1.56 ± 0.28  *  **  ## | 0.90 ± 0.25  # | *F* (4, 20) = 13.04 | < 0.0001 |

caspase3, cysteinyl aspartate specific proteinase 3; *F*, one-way analysis of variance test; H2M, 2 h/day intermittent hypobaric hypoxia preconditioned MCAO group; H6M, 6 h/day intermittent hypobaric hypoxia preconditioned MCAO group; HpM, persistent hypobaric hypoxia preconditioned MCAO group; Rabep1, Rabaptin, RAB GTPase binding effector protein 1; Hspa2, Heat shock protein family A (Hsp70) member 2; Chmp1a, Charged multivesicular body protein 1A; Arpc5, Actin related protein 2/3 complex, subunit 5 ; *p*, *p-*value of analysis of variance test; S, sham; SD, standard deviation

*: *p* < 0.05 vs M group **: *p* < 0.05 vs H2M group #: *p* < 0.05 vs H6M group ##: *p* < 0.05 vs HpM group

Supplementary Table 7. Clathrin and EEA1 positive cell ratio in immunofluorescence staining in ischemic rats

| **Positive cell ratio (%)** | **Groups** | | | | | **Statistical indicators** | ***p*** |
| --- | --- | --- | --- | --- | --- | --- | --- |
|  | **S** | **M** | **H2M** | **H6M** | **HpM** |  |  |
| n | 5 | 5 | 5 | 5 | 5 |  |  |
| Clathrin (Median, IQR) | 10.48  (5.50, 21.65)  *  #  ## | 38.67  (33.33, 44.44)  ** | 22.41  (17.02, 30.68)  * | 42.45  (17.54, 50.00) | 34.38  (27.93, 40.48) | Kruskal-Wallis test = 25.62 | < 0.0001 |
| EEA1 (Median, IQR) | 0.77  (0.00, 1.42)  * | 20.34  (15.56, 33.85)  **  # | 2.13  (0.00, 10.83)  * | 1.03  (0.00, 5.63)  * | 4.44  (0.00, 13.13) | Kruskal-Wallis test = 27.89 | < 0.0001 |

EEA1, Early endosome antigen 1; *F*, one-way analysis of variance test; H2M, 2 h/day intermittent hypobaric hypoxia preconditioned MCAO group; H6M, 6 h/day intermittent hypobaric hypoxia preconditioned MCAO group; HpM, persistent hypobaric hypoxia preconditioned MCAO group; IQR, interquartile range; M, middle cerebral artery occlusion (MCAO) group; NeuN, neuron-specific nuclear protein; *F*, ANOVA Test Statistic; *p*, *p-*value of ANOVA; S, sham; SD, standard deviation;

*: *p* < 0.05 vs M group **: *p* < 0.05 vs H2M group #: *p* < 0.05 vs H6M group ##: *p* < 0.05 vs HpM group
