# Supplementary figures and images for: Preconditioning With Intermittent Hypobaric Hypoxia Attenuates Stroke Damage and Modulates Endocytosis in Residual Neurons
Source: Front Neurol. 2021 Dec 15;12:750908. doi: 10.3389/fneur.2021.750908 (PMC8715922; doi:10.3389/fneur.2021.750908)

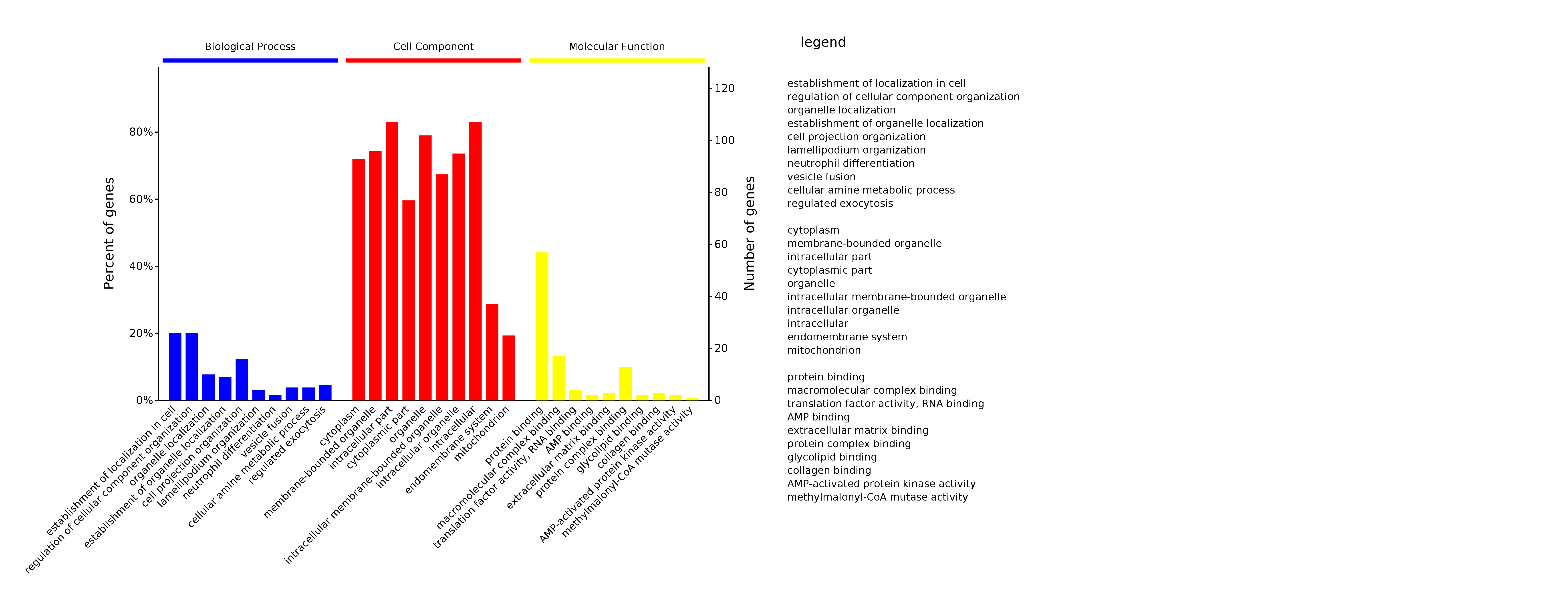

Supplement: Supplementary file 1 [file Data_Sheet_1.ZIP › supplementary data and figures/Proteomics/figures ú¿proteomicsú⌐/all_go_bar.png]

H6M vs M Volcano Plot

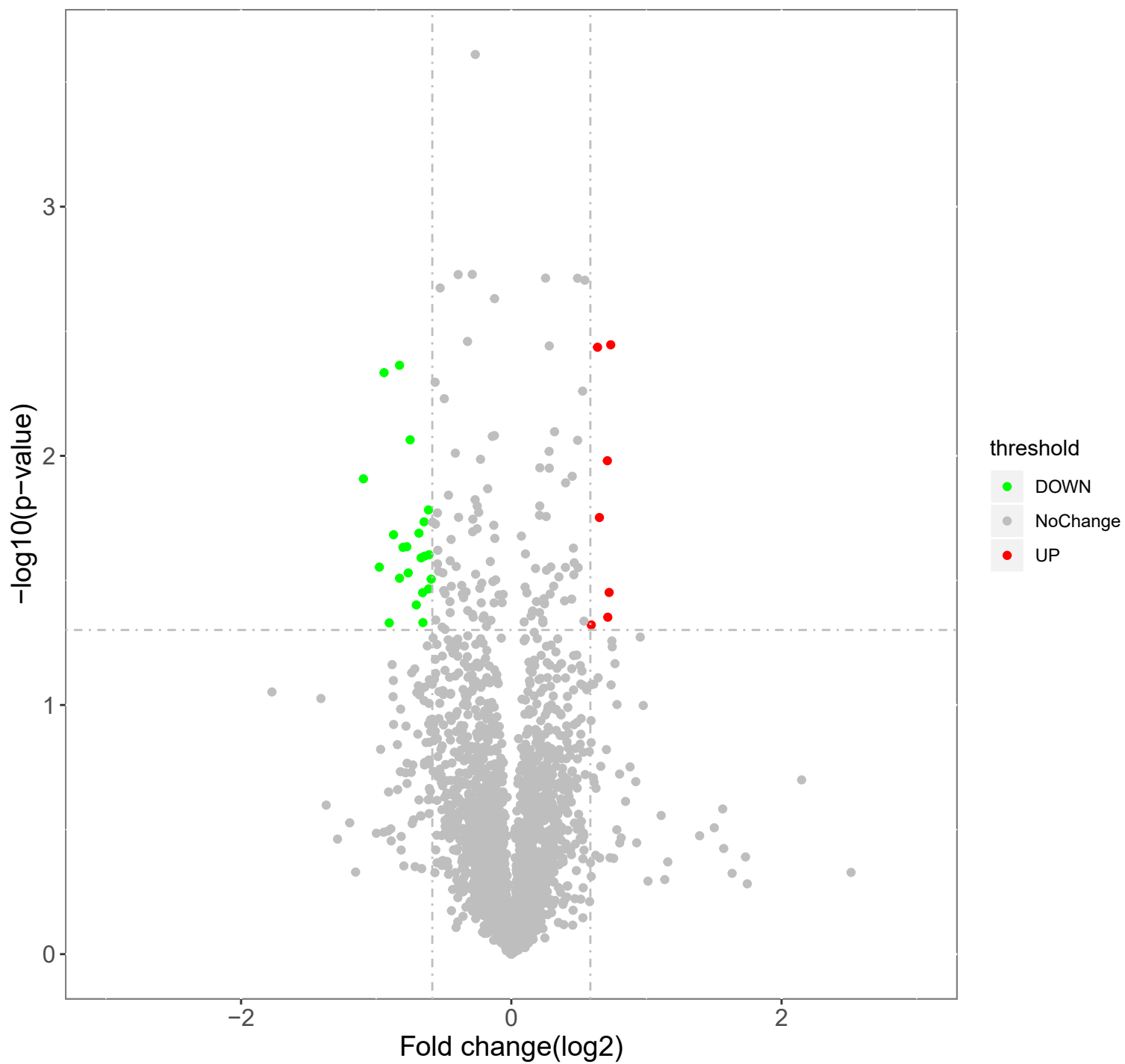

Supplement: Supplementary file 1 [file Data_Sheet_1.ZIP › supplementary data and figures/Proteomics/figures ú¿proteomicsú⌐/H6M vs M Volcano Plot.pdf]

# H6M vs M Heatmap

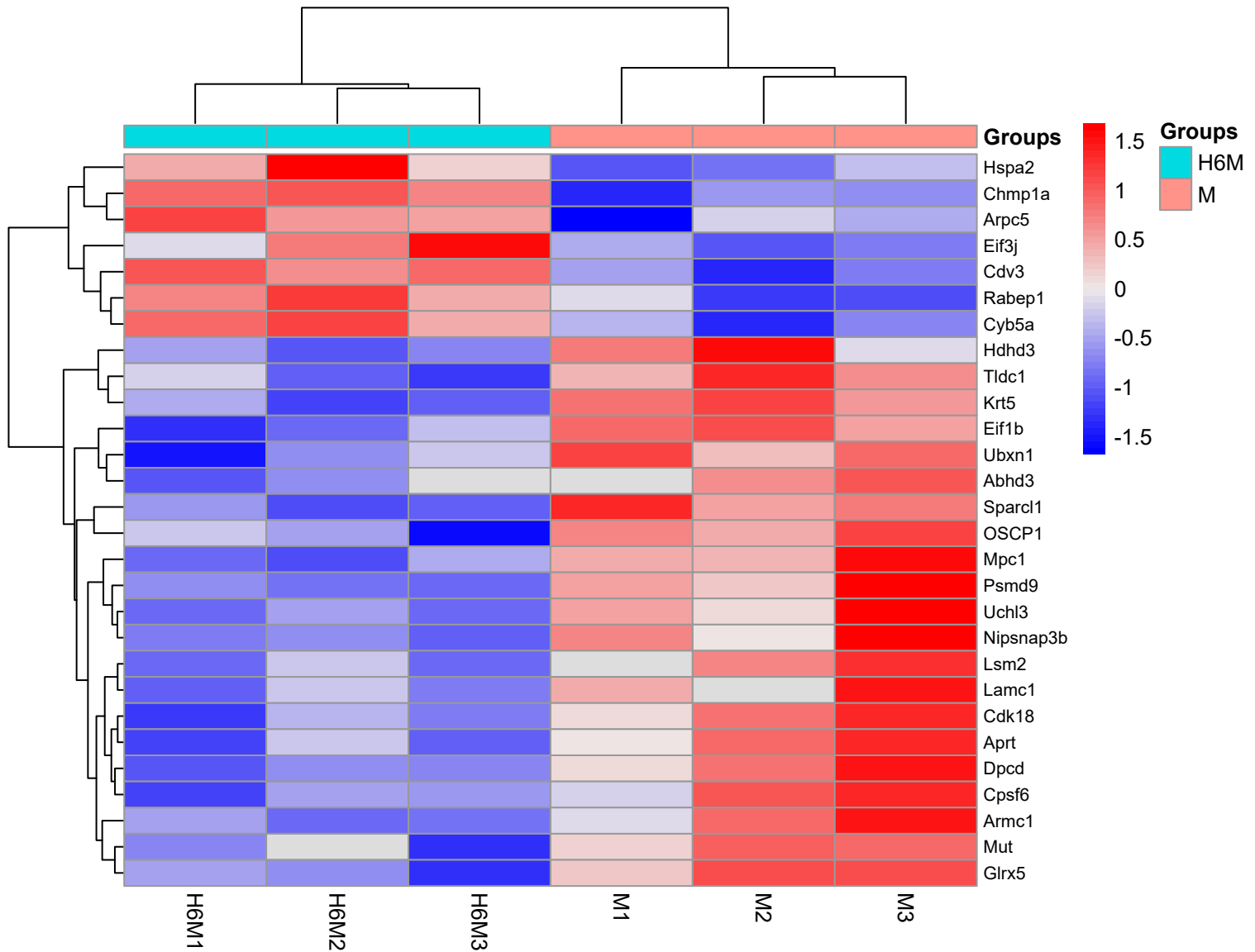

Supplement: Supplementary file 1 [file Data_Sheet_1.ZIP › supplementary data and figures/Proteomics/figures ú¿proteomicsú⌐/Heatmap-H6M vs M.pdf]

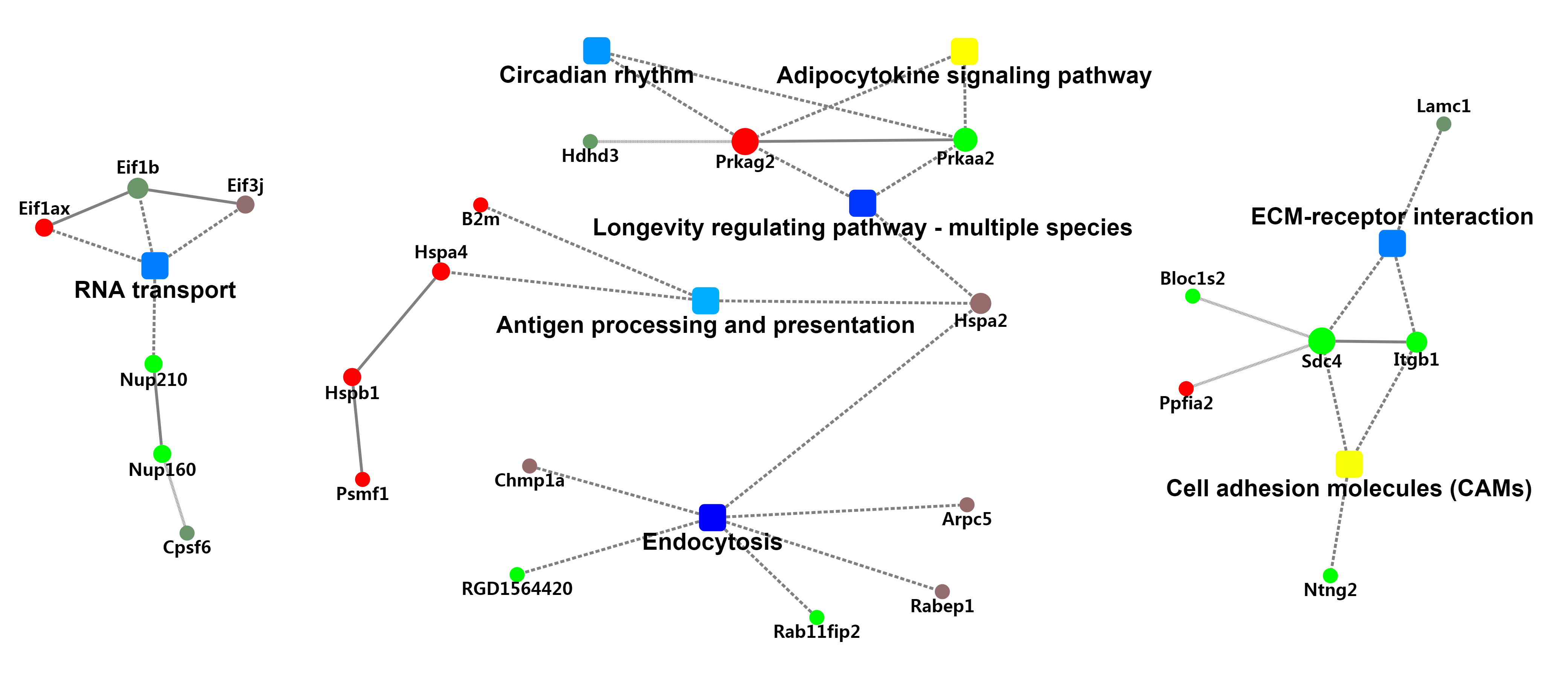

Supplement: Supplementary file 1 [file Data_Sheet_1.ZIP › supplementary data and figures/Proteomics/figures ú¿proteomicsú⌐/HM vs M PPI Overview.png]

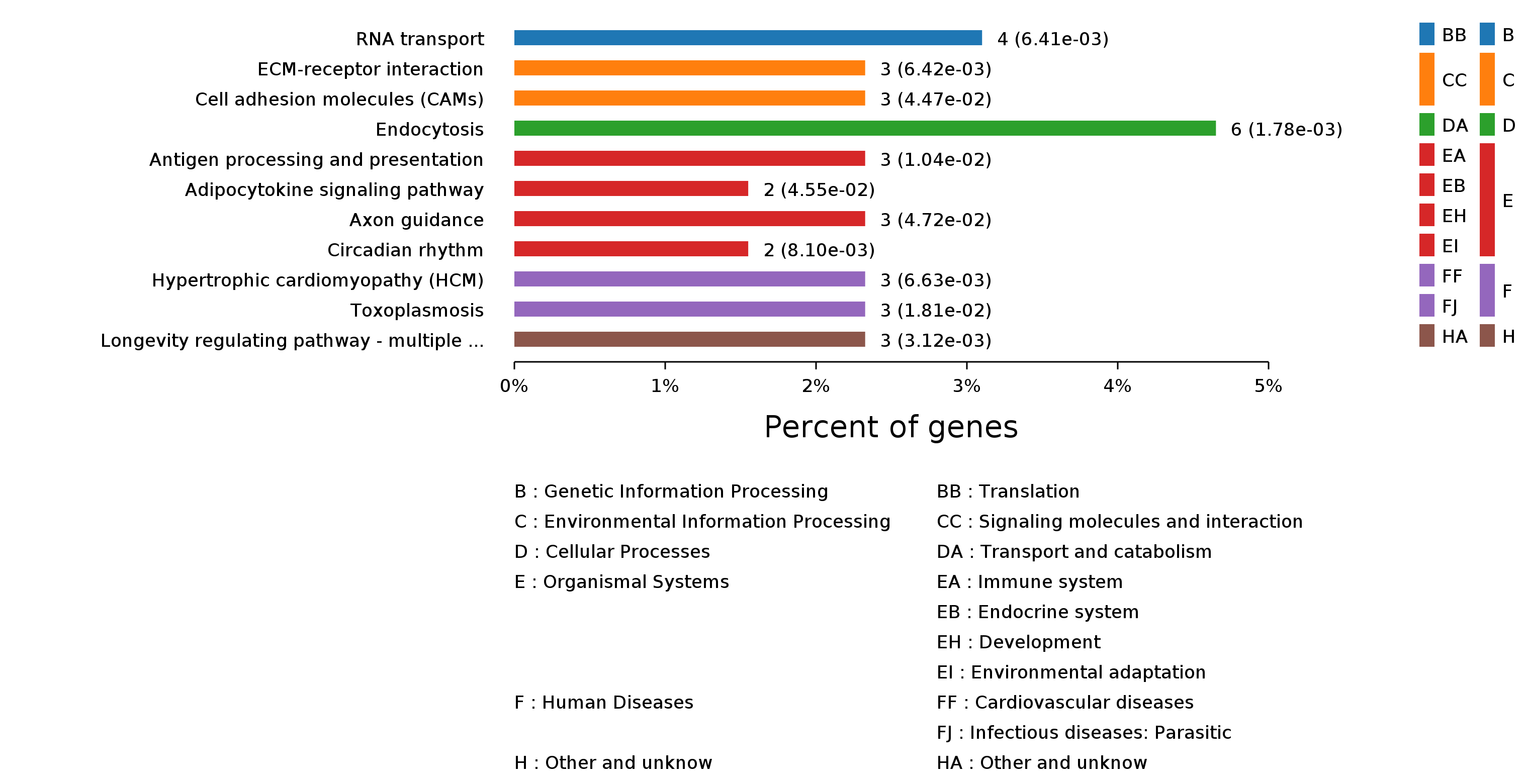

Supplement: Supplementary file 1 [file Data_Sheet_1.ZIP › supplementary data and figures/Proteomics/figures ú¿proteomicsú⌐/kegg_class.png]

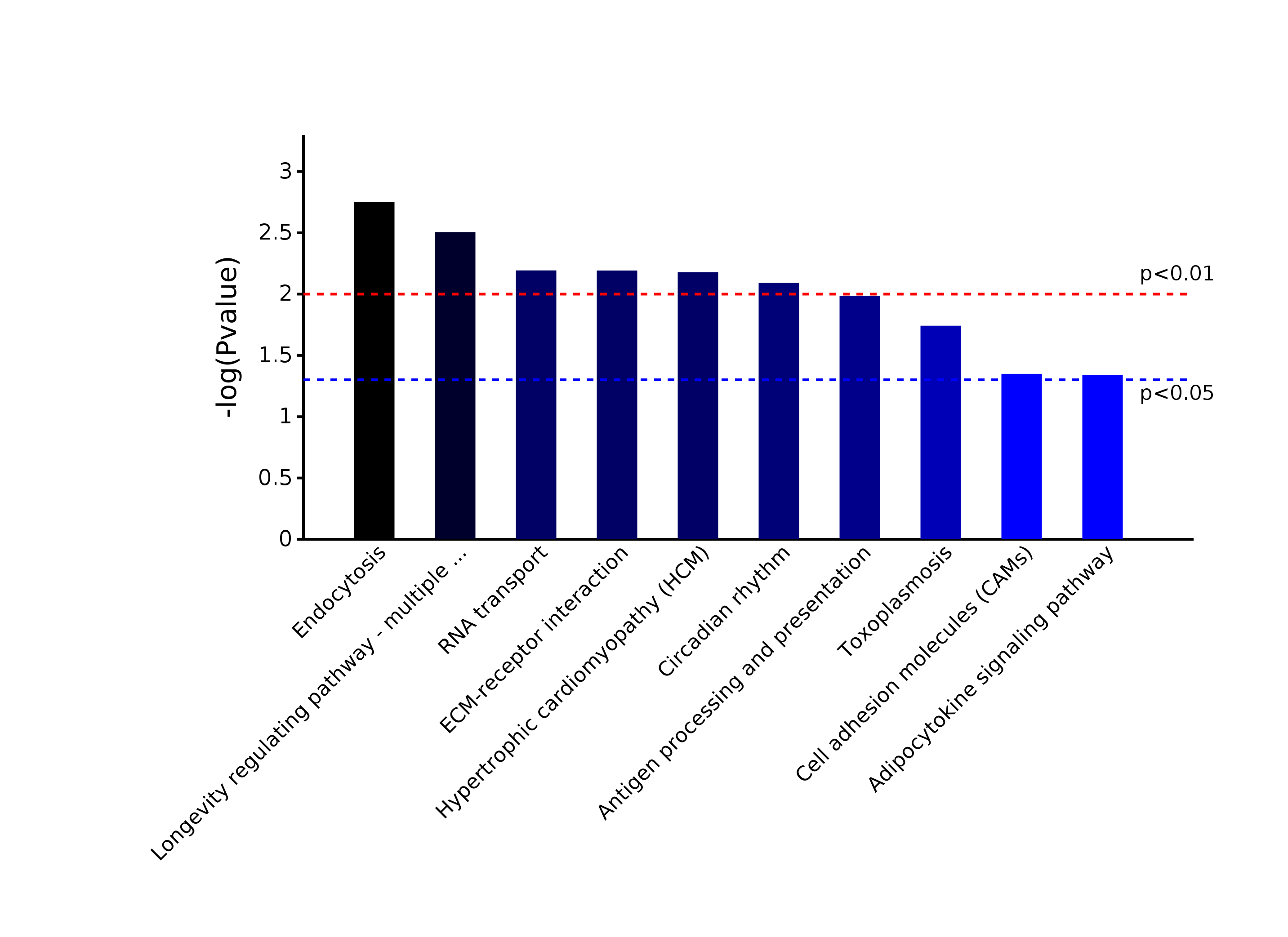

Supplement: Supplementary file 1 [file Data_Sheet_1.ZIP › supplementary data and figures/Proteomics/figures ú¿proteomicsú⌐/kegg_pvalue.png]

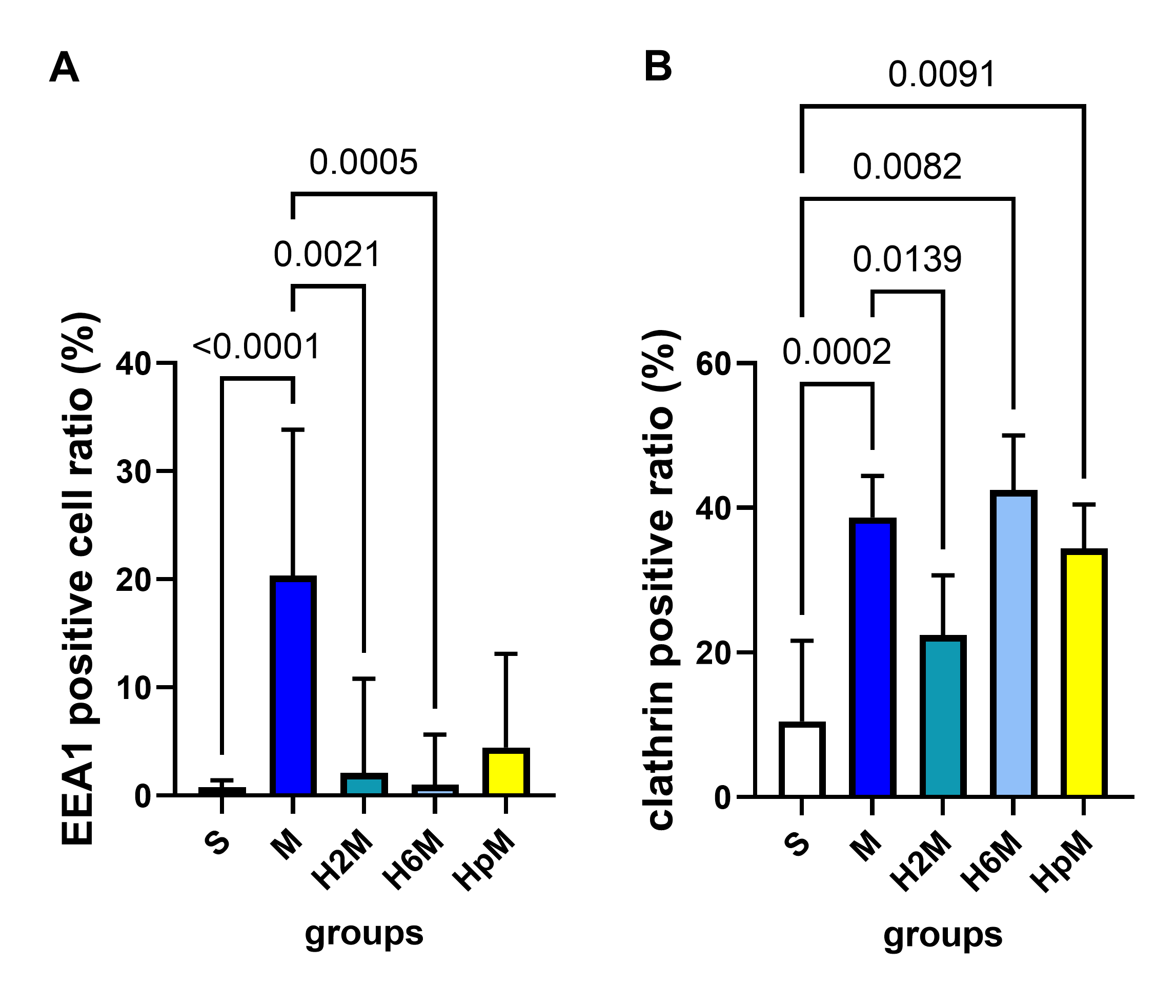

Supplement: Supplementary file 1 [file Data_Sheet_1.ZIP › supplementary data and figures/Supplemental Figure/supplementary Figure 1.tif]
